# Design of the Indian NCA study (Indian national collaboration on AIDS): a cluster randomized trial to evaluate the effectiveness of integrated care centers to improve HIV outcomes among men who have sex with men and persons who inject drugs in India

**DOI:** 10.1186/s12913-016-1905-5

**Published:** 2016-11-14

**Authors:** Sunil S. Solomon, Gregory M. Lucas, David D. Celentano, Allison M. McFall, Elizabeth Ogburn, Lawrence H. Moulton, Aylur K. Srikrishnan, M. Suresh Kumar, Santhanam Anand, Suniti Solomon, Shruti H. Mehta

**Affiliations:** 1 Johns Hopkins School of Medicine, 1830 E Monument St, Baltimore, MD 21205 USA; 2 Johns Hopkins Bloomberg School of Public Health, 615 N. Wolfe Street, Baltimore, MD 21205 USA; 3 YR Gaitonde Centre for AIDS Research and Education (YRGCARE), Chennai, India

**Keywords:** HIV/ADS, Men who have sex with men, People who inject drugs, India

## Abstract

**Background:**

Globally, men who have sex with men and people who inject drugs remain disproportionately affected by HIV, but they have not been the focus of prevention and treatment interventions in many resource-limited settings.

**Methods/Design:**

This cluster-randomized trial (conducted from June 2012 to June 2017), evaluates whether single-venue, integrated delivery of core HIV services to vulnerable high-risk populations improves service utilization and consequently, HIV testing and other outcomes along the HIV care continuum. Core services include: HIV counseling and testing, information, education and communication, condom distribution, needle and syringe exchange programs, opioid agonist therapy, management of sexually transmitted infections, tuberculosis screening, diagnosis, and treatment, and antiretroviral therapy. Stratified restricted randomization was used to allocate 22 Indian cities (10 men who have sex with men and 12 people who inject drugs sites) at a 1:1 ratio to either the intervention or control condition. Integrated care centers were scaled-up and implemented in the 11 intervention cities and outcomes will be assessed by pre- and post-intervention surveys at intervention and control sites. As men who have sex with men and people who inject drugs are hidden populations, with no sampling frame, respondent-driven sampling will be used to accrue samples for the two independent cross-sectional surveys.

**Discussion:**

For an AIDS-free generation to be realized, prevention, care and treatment services need to reach all populations at risk for HIV infection. There is a clear gap in access to services among men who have sex with men and people who inject drugs. Trials need to be designed to optimize utilization of services in these populations.

**Trial registration:**

ClinicalTrials.gov Identifier: NCT01686750

Date of Registration: September 13, 2012

**Electronic supplementary material:**

The online version of this article (doi:10.1186/s12913-016-1905-5) contains supplementary material, which is available to authorized users.

## Background

### Study rationale

India has an estimated 2.3 million HIV-infected persons [1]. The HIV epidemic in India has historically been driven by heterosexual transmission [1] with the exception of the Northeast where injection drug use is the primary driver [2–4]. Recent evidence suggests that the HIV epidemic in India has stabilized and may even be declining [1, 5] attributable to reductions in HIV prevalence among high-risk heterosexual populations (e.g., female sex workers, truck drivers, women attending antenatal clinics) who have been targeted by interventions since the mid 1980s. By contrast, men who have sex with men (MSM) and people who inject drugs (PWID) have not been targeted by interventions until recently, and represent two key populations with highest HIV burden in India currently [1, 6, 7].

The National AIDS Control Organization (NACO), India has estimated there are 2.35 million ‘high-risk’ MSM living in India [8]; however, estimates of prevalence of same-sex behavior among men are as high as 9 %, translating to as many as 45 million MSM [9, 10]. As in several other resource-limited settings, anal intercourse is a crime punishable by law in India [11]. Consequently, most MSM remain hidden and frequently marry women to conceal their MSM behavior [12]. HIV prevalence among MSM ranges between 7 and 41 %, with cities in the south reporting higher burden of infection [13–18].

India is home to the largest number of opiate users in the world (~3 million) [19] and ~ 1.1 million PWID [20, 21]. Historically, drug use in India was described in the Northeastern states due to their proximity to the ‘Golden Triangle’ – Burma, Laos, Thailand and Vietnam. Later, reports of injection drug use emerged from large metropolitan cities across India [22–25] and more recently, reports of increases in drug use have been reported from cities in the Northwestern states of India [26–29], which border the ‘Golden crescent’ – Afghanistan and Pakistan. HIV prevalence among PWID ranges from 10 to 30 %, with cities from the Northeastern states bearing a higher burden of infection [3, 4, 23, 24, 30–32].

Although MSM and PWID together account for a minority of HIV cases in India, they are major drivers of the epidemic in some regions. MSM and PWID share commonalities that make HIV service delivery challenging: (1) both behaviors are punishable by law, discouraging people from seeking prevention and treatment services [11, 12]; (2) high levels of experienced stigma from the general and medical communities have been reported by both [33–35]; and (3) prevention and treatment services are only accessible via a fragmented service delivery model. All of these have contributed to service underutilization.

### Study aims and hypotheses

This trial evaluates whether single-venue, integrated delivery of core services to MSM and PWID improves service utilization and consequently HIV testing and other outcomes along the HIV care continuum. Core services recommended by the World Health Organization (WHO), United Nations Office on Drug Control (UNODC) and the Joint United Nations Programme on HIV/AIDS (UNAIDS) for PWID (several of which are also applicable to MSM) include: (1) HIV counseling and testing [HCT], (2) information, education and communication (IEC), (3) condom distribution, (4) needle and syringe exchange programs [NSEP], (5) opioid agonist therapy [OAT] for opioid users, (6) management of sexually transmitted infections [STIs], (7) viral hepatitis vaccination, (8) tuberculosis [TB] diagnosis, prevention and treatment, and (9) antiretroviral therapy [ART] [36]. Further, WHO recommends these services be delivered in an affordable, accessible and non-discriminatory manner. Together these interventions can improve awareness of HIV status, reduce injection-related and same-sex risk behavior, improve health, reduce mortality, and decrease the infectiousness of HIV-infected persons in the community through effective viral suppression. We will evaluate the community-level effectiveness of MSM/PWID-focused integrated care centers (ICCs), which will provide core and group-specific HIV prevention and treatment services at a single venue utilizing a cluster-randomized trial design in 22 sites across India.

Our hypotheses are:

1. Establishing ICCs will increase access to HCT and knowledge of HIV status among MSM and PWID.
2. In the subset of HIV-infected MSM and PWID, ICCs will increase access to care, use of ART and will decrease community viral load.
3. Establishing ICCs will decrease transmission risk behaviors and HIV incidence among MSM and PWID via improved access to prevention services (NSEP, OAT, IEC).

## Methods/Design

### Study design

**Fig. 1:**
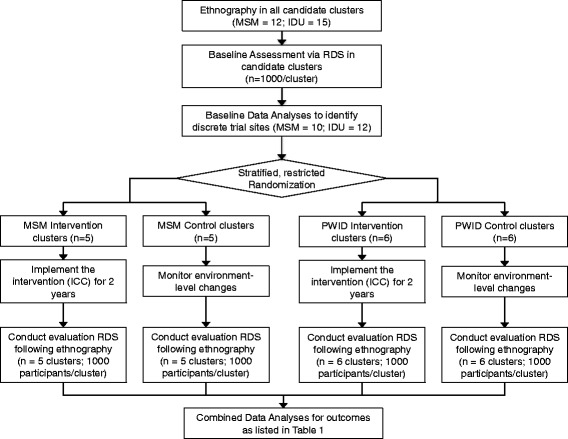
Study Design. Abbreviations: MSM, Men who have sex with men; PWID, People who inject drugs; RDS, Respondent-driven sampling; ICC, Integrated care centers

The study design is a cluster-randomized trial with parallel MSM and PWID strata. ICCs were scaled-up and implemented at sites allocated to the intervention arm, and outcomes will be assessed by pre- and post-intervention surveys at intervention and control sites (Fig. 1). As MSM and PWID are hidden populations with no sampling frame, respondent-driven sampling (RDS) will be used to accrue samples for the two independent cross-sectional surveys. Sites (10 MSM and 12 PWID sites) were randomized in a 1:1 allocation ratio. The trial is currently underway. The pre-intervention survey took place between September 2012 and December 2013; the ICCs were scaled up between June 2014 and February 2015. Post-intervention survey commenced in August 2016 and is expected to be complete by April 2017.

### Study sites

**Fig. 2:**
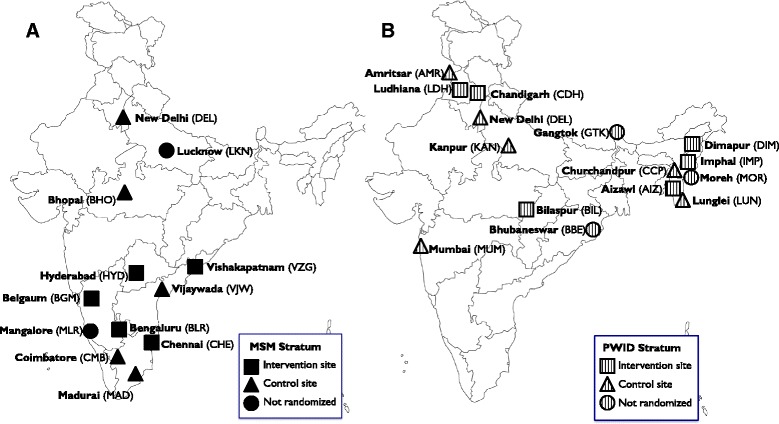
Study Sites. Panel **a**. MSM sites represent cities with established HIV epidemics among MSM (Chennai, Hyderabad, Bengaluru), smaller cities in high HIV prevalence states (Coimbature, Madurai, Vishakapatnam, Vijaywada, Mangalore, Belgaum) and cities with anecdotal reports of HIV among MSM but no published reports (New Delhi, Bhopal, Lucknow). Panel **b**. PWID sites represent cities with established drug use epidemics (Aizawl, Churchandpur, Dimapur, Gangtok, Imphal, Lunglei, Moreh), large cities (New Delhi, Mumbai) cities with documented emerging drug use epidemics (Amritsar, Chandigarh, Ludhiana) and cities with anecdotal evidence of emerging drug use epidemics (Bilaspur, Bhubaneswar, Kanpur). Note New Delhi has two control sites (one for the MSM stratum and one for the PWID stratum)

Twenty-seven candidate sites were selected (15 PWID, 12 MSM; Fig. 2) of which 22 were included in the trial. Sites were selected to represent regions of India where there was preliminary evidence of high HIV prevalence or high risk of HIV acquisition through discussions with the Targeted Interventions (TI) Division of NACO, India. We ensured sufficient distance between sites to minimize potential overlap (contamination) between intervention and control sites. MSM sites were selected to represent cities with established HIV epidemics among MSM, smaller cities in high prevalence states and cities with anecdotal reports of HIV among MSM but no published reports [1]. PWID sites were selected to represent varying stages of drug use epidemics (established drug use epidemics, large metropolitan cities, cities with documented emerging drug use epidemics and cities with anecdotal evidence of emerging drug use) [1].

### Design of the intervention

The intervention is vertically integrated delivery of HIV prevention and treatment services in stand-alone venues – called ICCs–to disenfranchised high-risk groups. These centers will provide services critical to HIV prevention and essential outpatient services for HIV-infected persons. The core services at MSM ICCs are: HCT, condom promotion and distribution, diagnosis and treatment of STIs, testing services for spouses, HIV and risk reduction counseling services as well as counseling for substance use and depression. In addition, PWID ICCs will also include NSEP and OAT (using medically managed buprenorphine). Finally, in both PWID and MSM ICCs, we established linkages with local government centers to deliver treatment for HIV (ART) and tuberculosis. All ICC services are currently available in India but are typically provided by independent and insulated service organizations. The keystone of the ICC intervention is vertically integrated service delivery to disenfranchised high-risk groups.

ICCs are based on the premise that interventions that combine approaches and address multiple levels (e.g., community-, network-, and individual-levels) can lead to sustainable change [37–42]. At the core of our model is a structural intervention that seeks to bring about *community-level* change through provision of integrated services in a non-discriminatory setting with nested approaches guided by social and behavioral science theories to induce behavioral change at *network* and *individual* levels. The structural change (i.e., establishment of ICCs) is based on the Andersen and Aday model of health care utilization [43, 44]. The goal is to address environmental, predisposing, enabling, and need factors in MSM and PWID that promote or inhibit health services use. To implement this structural change, we rely on the diffusion of innovations [45] and ‘tipping point’ theories [46]. We propose to leverage MSM and PWID social networks to disseminate information about the ICCs to improve ICC utilization [47]. Once participants visit these centers, we target individual-level behavior change according to the Information, Motivation and Behavior (IMB) Skills theoretical framework [48, 49].

### Control condition

In control sites, all services described above will be locally available. They are provided by the government free-of-charge, but delivered in segregated centers that cater to both the general population as well as key populations (i.e., there are no MSM or PWID specific centers currently in any of the control cities). HCT is delivered by government Integrated Counseling and Testing Centers (ICTC) and private laboratories. ART is delivered through government ART centers. Tuberculosis care is delivered through government Directly Observed Therapy (DOT) centers. STI testing and treatment is provided by government hospitals. For PWID, NSEP and OAT are available free of charge but delivered through different venues. While OAT is predominantly delivered in the government sector (except in the Northeast where non-governmental organizations [NGOs] deliver OAT), NSEP is almost exclusively delivered by NGOs.

### Study outcomes

The primary and secondary study outcomes are listed in Table 1. They will be assessed by both objective laboratory data and self-reported data from behavioral and medical surveys. Our primary outcome is HCT in the prior 12 months. Participants will be asked about HIV testing in two ways. First, they will be asked whether they have ever had an HIV test and if so then to recall the last time they had an HIV test. Interviewers have been extensively trained on capturing an accurate response to this question. Participants are asked if they recall the exact date that they were tested. If so then the date is captured. If unaware of the exact date, interviewers have been trained to work with the participant to arrive at an approximate time frame by using personal events such as birthdays and anniversaries as well as cultural and religious events such as Christmas and Diwali. Second, persons who report that they have not been tested for HIV or that they tested negative at their last test are also asked if they have ever been told by a health care professional that they are HIV positive and to recall the last time they were told that they were HIV positive. A response to each of these questions is mandatory. In calculating the outcome variable, persons who report being HIV positive and being tested more than 12 months ago will be excluded from the denominator. Persons will be considered to have the outcome of interest if they report either having had an HIV test in the prior 12 months or being told by a health care professional that they were HIV positive in the past 12 months.

**Table 1:** Outcome definitions for the Indian National Collaboration on AIDS (NCA) study

	Outcome	Data inputs	Population (denominator)	Definition
Primary Outcome				
HIV testing				
1	HIV testing in the prior 12 moths	Survey	All participants, excluding those that report being HIV-positive AND report being tested more than 12 months ago.	Either 1 OR 2: 1. Reports HIV test within the prior 12 months OR 2. Was told he/she had HIV within the last 12 months
Secondary Outcomes				
Awareness of HIV status, access to care, and HIV treatment				
2	Awareness of HIV status among HIV-seropositive persons	1. Survey 2. Serologic HIV test	HIV seropositive participants	Reports having a positive HIV test OR being told by a doctor that he/she had HIV.
3	Accessing HIV medical care in prior 6 months	1. Survey 2. Serologic HIV test	HIV seropositive participants	Reports seeing a doctor about HIV AND reports visit with the indicated doctor in the prior 6 months.
4	Use of ART among ART-eligible	1. Survey 2. Serologic HIV test	HIV seropositive participants who meet either criteria 1 or 2 1. Reports taking ART at any time in the past OR 2. CD4 < 350 cells/μL	Reports ART use in prior 30 days.
5	Use of trimethoprim-sulfamethoxazole (TMP-SMX) when indicated	1. Survey 2. Serologic HIV test 3. CD4	HIV seropositive AND CD4 count < 200 cells/μL	Reports taking TMP-SMX in past 30 days
6	Viral suppression among ART-eligible	1. Survey 2.Serologic HIV test 3. CD4 4. HIV RNA	HIV seropositive participants who meet either criteria 1 or 2 1. Reports taking ART at any time in the past OR 2. CD4 < 350 cells/μL	HIV RNA <150 c/mL
7	Viral suppression among HIV-positive	1. Serologic HIV test 2. HIV RNA	HIV seropositive participants	HIV RNA <150 c/mL
8	Average CD4 cell count among HIV-positive	1. Serologic HIV test 2. CD4 cell count	HIV seropositive participants	CD4 cell count
Risk behaviors, substance use, and depression				
9	Unprotected anal intercourse with non-main male partner in prior 6 months [MSM]	Survey	Participants at MSM sites	Does not report “always” using a condom during insertive or receptive anal sex with non-main (e.g., casual, one-time partner, sex worker) male partners in the prior 6 months
10	Number of non-main male sexual partners in prior 6 months [MSM]	Survey	Participants at MSM sites	Number of non-main male (e.g., casual, one-time partner, sex worker) male partners with whom the participant reports having insertive or receptive anal sex in the prior 6 months
11	Symptoms of sexually transmitted infection [MSM]	Survey	Participants at MSM sites	Reports genital/anal discharge, pain, or ulcer in prior 6 months
12	Syphilis infection	1. RPR test 2. TPHA test	Participants at MSM sites	Positive for syphilis infection by both RPR and TPHA tests
13	Shared injection equipment in prior 6 months [PWID]	Survey	Participants at PWID sites	Reports sharing (passing or receiving) a needle and/or syringe with another individual in the prior 6 months
14	Shared injection equipment at last use among active injectors [PWID]	Survey	Participants at PWID sites that report injection of one or more drugs in prior 6 months	Reports sharing (passing or receiving) a needle and/or syringe with another individual at last injection
15	Reported injection abstinence in prior 6 months [PWID]	Survey	Participants at PWID sites	Denies injecting any drug in prior 6 months
16	Hazardous alcohol use or dependence	Survey	All participants	Score ≥8 (hazardous) or ≥15 on Alcohol Use Disorder Identification Test (AUDIT) [61]
17	Depression	Survey	All participants	Score ≥ 10 on Patient Health Questionnaire (PHQ)-9 [62]
Services and stigma				
18	Spouse HIV testing among married participants	Survey	Participants who report being married	Reports spouse has ever been tested for HIV
19	Symptoms of sexually transmitted infection for which participant sought care in prior 6 months [MSM]	Survey	Participants at MSM sites	Reports genital/anal discharge, pain, or ulcer in prior 6 months AND reports seeking medical care for symptom(s)
20	Used needle/syringe exchange program (NSEP) in prior 6 months [PWID]	Survey	Participants at PWID sites	Reports NSEP use in prior 6 months
21	Used needle/syringe exchange program (NSEP) in prior 6 months among active injectors [PWID]	Survey	Participants at PWID sites that report injection of one or more drugs in prior 6 months	Reports NSEP use in prior 6 months
22	Used opioid agonist therapy (OAT) in prior 6 months [PWID]	Survey	Participants at PWID sites	Reports OAT in prior 6 months
23	Stigma subtypes	Survey	All participants	Summed score from each of four 6-item stigma scales (enacted, vicarious, felt normative, and internalized stigma) [63]
Community viral load and HIV incidence				
24	Prevalence of viremic individuals in population	1. Serologic HIV test 2. HIV RNA	All participants	Prevalence of HIV-positive subjects with HIV RNA >150c/mL [64]
25	Average viral load in HIV-positive participants	1. Serologic HIV test 2. HIV RNA	HIV seropositive participants	Average (log10) HIV RNA
26	HIV incidence	1. Serologic HIV test 2. HIV RNA 3. CD4 cell count 4. BED assay 5. Avidity index	Participants who meet criteria 1 or 2 1) HIV-seronegative OR 2) HIV-seropositive participants who meet criteria for recent infection by HIV RNA, CD4, BED assay, and avidity assay	Cross-sectional HIV incidence estimated as described previously [6, 7, 64]

### Implementation of the trial

#### Ethnography

Before initiating the baseline assessment, we conducted ethnographic research and community preparedness in 27 candidate study sites to: (1) identify potential “seeds” for RDS; (2) assess potential for contamination across study sites by exploring mobility; and (3) understand regional variation in existing HIV prevention and treatment services. On average, two focus group discussions (FGDs) and 6-8 in-depth interviews (IDIs) were conducted in each potential study site.

During this preparatory phase, we also conducted community meetings for a 3-month period with peer leaders and outreach workers from the MSM/PWID NGOs in the community to inform them of the study. RDS is a peer-driven chain referral strategy that hinges on the ability of participants to recruit peers. Injection drug use and same-sex behavior are both stigmatized in India and punishable by law. Thus, a key goal of these community meetings was to make the target populations aware that they might receive coupons from their peers/friends/sexual or injection partners to participate in a study and that this study was not a ploy to harm or arrest MSM or PWID but rather to evaluate their access to HIV services and understand the needs of the communities.

#### Baseline pre-intervention assessment

The goal of the baseline assessment, conducted between September 2012 and December 2013, was to establish baseline prevalence of study outcomes for sites in the trial. We accrued samples of ~1000 eligible participants in each candidate study site where we partnered with one or more NGOs working with the target population. Sampling was conducted using RDS, a chain-referral sampling method [50, 51] which approximates a probability sample of populations when sampling frames are lacking. It is similar to snowball sampling [52] except the recruitment process is implemented in a systematic way that allows for the calculation of selection probabilities. Participants select and provide referral coupons to individuals in their peer network [50]. Verbal informed consent was obtained from all participants.

Eligibility criteria were:

1. ≥18 years of age
2. Present a valid RDS referral coupon
3. Be able to comprehend one of the languages in which the survey would be available
4. Male gender (MSM)
5. Oral or anal intercourse with another man in the prior year by self-report (MSM)
6. History of drug injection in the prior two years by self-report (PWID)

RDS was initiated at each site with participants (“seeds”) selected during ethnography. Two seeds were selected from a list of 10 per site to represent varying demographic, geographic (different parts of the city), HIV status, and for MSM sites, sexual identity (insertive vs. penetrative vs. versatile) and for PWID sites, drug-related diversity (e.g., heroin vs. other opioid injection; daily vs. less frequent injection) in the local population. While the initial plan was to select 4–7 seeds, recruitment in nearly all sites proceeded at a rapid pace – therefore, in 25 of 27 sites no additional “seeds” were added. In one MSM and one PWID site, a third seed was added as recruitment was slower than the other sites. In one PWID site (Moreh), recruitment was terminated prematurely for safety considerations due to civil unrest.

“Seeds” and subsequent RDS recruits were asked to provide a scan of their fingerprint. The fingerprint image is immediately converted to a unique hexadecimal code and stored; the image itself is not stored. This code is linked to a study ID, which is used on participant forms and laboratory samples and is also used to rule out duplicate enrollments within a site and to link participation across multiple phases of the study.

All participants underwent a survey followed by HIV pre- and post-counseling and a blood draw. Survey modules and laboratory tests are provided in Table 2. English language versions of the surveys used at MSM and PWID sites are available as Additional files 1 and 2. The survey was conducted by trained interviewers who were hired expressly for the pre- or post-intervention RDS surveys, did not work with or have previous interactions with the target population in question, and had no stake in the outcome of the ICC evaluation. Interviewers recorded answers to a web-based, secure database via laptops and a local area network. RDS interviewers and support staff were trained extensively on visit flow, documentation, questionnaires, and laboratory procedures using a single training protocol across sites. Quality control for the survey was maintained by programmed logic checks and real-time data monitoring algorithms and by site supervisors who monitored 5 % of randomly selected interviews for proper interviewing technique. Constraints placed on the database required interviewers to answer every question on the survey and ensured that missing data was minimal.

**Table 2:** Data collection

		MSM	PWID
Survey modules			
Demographics		x	x
Peer network size		x	x
HIV testing, care and medications (HIV care continuum)		x	x
HIV treatment knowledge (including questions on other local HIV testing and treatment efforts)		x	x
Substance use (drugs, alcohol), injection-related risk behavior, sexual risk behavior		x	x
Service utilization (NSEP, OAT, condom provision)		x	x
Tuberculosis history		x	x
Depressive symptoms (Patient Health Questionnaire-9 [62, 65])		x	x
Social support		x	x
Stigma (Enacted, vicarious, felt normative, internalized MSM stigma)		x	
Stigma (Enacted, vicarious, felt normative, internalized PWID stigma)			x
Quality of life (adapted version of EuroQOL [66])		x	x
Acceptability of novel prevention interventions (early ART, circumcision, PrEP)		x	x
Sexual health (including STI history)		x	
Hepatitis C virus and Hepatitis B virus testing, care and treatment			x
Laboratory testing			
HIV^8^	Determine HIV 1/2, Alere Medical Co., Ltd., Chiba, Japan First Response HIV Card Test 1-2.0, PMC Medical India Pvt, Ltd, Daman, India Signal Flow Through HIV 1 + 2 Spot/Immunodot Test kit, Span Diagnostics Ltd, Surat, India	x	x
CD4 count^a^	Flow cytometry, Epics XL – MCL, Beckman Coulter Inc., USA	x	x
HIV RNA^a^	RealTime HIV-1 Assay, Abbott Laboratories, Abbott Park, Illinois, USA	x	x
BED assay^a^	Aware™ BED™ EIA HIV-1 Incidence Test (IgG Capture HIV-EIA), Calypte Biomedical Corporation, Portland, OR, USA	x	x
Avidity [67]^a^	GS HIV-1/HIV-2 PLUS O EIA, Biorad Laboratories, Redmond, USA using diethyl amine as the chaotropic agent	x	x
HSV-2	Anti-HSV-2 (gG2) ELISA (IgG), Euroimmun Medizinische Labordiagnostika AG, Lubeck, Germany	x	
Syphilis	RPR Test Kit, Span Diagnostics Ltd. Surat, India Immunotrep TPHA, Omega Diagnostics Limited, Scotland, UK	x	

Abbreviations: *NSEP* needle and syringe exchange programs, *OAT* opioid agonist therapy, *MSM* men who have sex with men, *PWID* people who inject drugs, *ART* antiretroviral therapy, *PrEP* pre-exposure prophylaxis, *STI* sexually transmitted infections

^a^Tests performed only among those who tested HIV positive. Cross-sectional estimation of HIV incidence was based on a multi-assay algorithm (MAA) validated for HIV Subtype C [68] – the predominant subtype in India that included CD4, HIV RNA, BED and Avidity [69]

Participants were also asked to recruit up to two members of their sexual (MSM) or drug-using networks (PWID) who satisfied the study eligibility criteria using bar-coded coupons that had a holographic image to hinder replication attempts. If participants successfully referred eligible participants, they received an additional incentive of INR 50 (USD 0.8) per eligible person recruited in addition to compensation of INR 250 (USD 4.1) for completing the pre-intervention assessment. Participants were recruited in successive RDS waves at each site until the desired sample size was accrued.

#### Randomization

Randomization took place after the pre-intervention assessment was completed. First, we selected 12 PWID sites (from 15) and 10 MSM sites (from 12) for randomization. In the PWID stratum, two sites were removed because of logistical challenges that deemed them unsuitable for randomization (Moreh, Gangtok). Three additional sites were dropped based on very low HIV prevalence (Bhubaneshwar [PWID-stratum], Lucknow and Mangalore [MSM-stratum]).

We used a restricted stratified randomization approach to randomly distribute the 22 sites to either the intervention or control condition [53]. In CRTs, the number of randomized units is relatively small and large imbalances between study arms may occur if randomization is unrestricted; hence, restricted randomization is often used to ensure reasonable balance between study arms in important factors. However, the desire for balance between arms must be balanced against leaving a sufficient number of randomization options (e.g., at least 100).

Sites were stratified based on risk group (MSM and PWID) and then additional restriction criteria were used to ensure balance, first within strata and then overall. Within strata, restrictions were made on the basis of geography, HIV prevalence and the primary outcome: HCT in the prior 12 months (Table 3). Additional restrictions were made on outcomes among HIV positive persons. All within-strata restrictions were based on RDS-weighted proportions of the outcomes. After strata-specific restrictions were made, the two strata were combined to derive a combined set of allocations. Final restrictions were made using the same outcomes with the exception that both RDS-weighted and unweighted proportions were considered.

**Table 3:** Description of stratum-specific and overall restriction criteria

	Stratum-specific		Overall
	MSM	PWID	
Geographical Restrictions			
Tamil Nadu (3)	3 sites distributed at a ratio of 2:1 (Madurai/Chennai in separate arms)		
Andhra Pradesh (3)	3 sites distributed at a ratio of 2:1		
Karnataka (2), Bhopal, Delhi	4 sites distributed with at least one site in each arm		
Northeast (5)		5 sites distributed at a ratio of 3:2	
North (4)		4 sites distributed at a ratio of 2:2	
West/Central India (3)		3 sites distributed at a ratio of 2:1	
Restrictions Based On Outcomes			
HIV prevalence	<1.5 %^a^	<2 %^a^	<2 %^c^
Percentage who had HIV test in the prior 12 months [PRIMARY OUTCOME]	<5 %^a^	<5 %^a^	<5 %^c^
Percentage of HIV positive aware of status	<10 %^a,b^	<10 %^a,b^	<10 %^c^
Percentage of HIV positive seen HIV provider in past 6 months	<10 %^a,b^	<10 %^a,b^	<10 %^c^
Percentage of HIV positive currently on antiretroviral therapy	<10 %^a,b^	<10 %^a,b^	<10 %^c^
Percentage of HIV positive with undetectable HIV RNA	<9 %^a^	<9 %^a^	<9 %^c^

^a^Restrictions placed on only RDS-weighted proportions; RDS-I estimator used for PREVALENCE and TEST; RDS-II estimator used for other outcomes because RDS-I estimator could not be calculated

^b^Restriction was across three outcomes (Proportion of HIV positive aware of status, Proportion of HIV positive seen HIV provider in past 6 months, Proportion of HIV positive currently on antiretroviral therapy); Only those with values >10 % in 2 of the three outcomes were excluded

^c^Restrictions placed on RDS-weighted and unweighted proportions

Restrictions related to geography and HIV prevalence were imposed because both were related to the stage of the HIV epidemic in the target population and HIV service availability. Further, geographic restrictions were important for political reasons, such that all intervention sites were not restricted to one region or state. Additional covariates used in the restriction process represented the primary and secondary outcomes. Of a total 232,848 possible allocations, 596 allocations remained after all restrictions. Across all combinations, there were only two sites that had >75 % chance of being randomized to the same arm [53]. Three individuals independent from the study who were blinded to the allocation sequence associated with each of the possible numbers chosen (e.g., 001 to 596) were asked to draw numbered balls from an opaque container to arrive at the final 3 digit number, corresponding to a numbered randomization combination. A recording of the randomization is available at: https://www.youtube.com/watch?v=vmHYHgv_uS0. The final allocation is shown in Fig. 2.

#### Intervention implementation

##### Venue selection

In each intervention site, venues were selected for scale-up following discussions between the State AIDS Control Society, NGO leaders, MSM/PWID community members and study investigators. In all cities, only one site was selected for scale up unless mobility within the city was restricted due to distance or unsafe circumstances, in which case more than one ICC was scaled-up (e.g., Imphal – 3, Chandigarh – 2, Bilaspur – 2). For the PWID sites, ICCs were distributed between the private sector (Imphal, Dimapur and Aizawl) and the government sector (Chandigarh, Ludhiana and Bilaspur). For the MSM sites, four ICCs were nested within existing government facilities – only the Hyderabad ICC was situated within an NGO. ICCs were scaled up between July 2014 and February 2015.

##### Service delivery

Core services (HCT, STI testing and treatment, condoms and individual and group counseling) are available on-site at the ICCs. PWID ICCs also provide daily OAT (7 days a week) and NSEP; in addition, most ICCs also conduct field-based NSEP using outreach workers from the ICCs. ART and anti-tuberculosis therapy (ATT) are operating through a link model, in which medications and testing support are provided through government centers, but service delivery and patient follow-up takes place at ICCs. All pre-treatment work-up (e.g., CD4, clinical examination, etc.) is performed at the ICC prior the patient’s referral to the government center to initiate ART. Following the initial visit to initiate ART at the government ART center, the patient is able to collect his/her monthly refills from the ICC (the “link”). He/she undergoes monitoring, monthly medication dispensing and clinical exams, as required, at the ICC and results are provided semi-annually to the government ART centers to update their records. Thus, the patient will only have to visit the government ART center once for registration and semi-annually thereafter. For each visit to the government center, participants are accompanied by an ICC peer-health worker.

For tuberculosis care, samples for screening and diagnosis are collected at the ICC, with confirmatory testing taking place at government centers. If a participant is diagnosed with TB, he/she is linked to the government DOT center most convenient to him/her for treatment initiation and follow-up. There are over 400,000 DOT centers in India ensuring easy access to all populations with excellent retention rates. Peer-health workers from the ICCs follow up with clients diagnosed with TB to ensure completion of the DOT program.

##### Client tracking

Peers and community health workers are responsible for tracking clients (via telephone and home/field visits) with respect to use of HIV services. Those who receive an HIV test and tested HIV negative are tracked within one year for repeat testing. Those who are HIV positive and not yet eligible for ART (CD4 > 350 cells/μl) are tracked if they miss a quarterly visit with the on-site clinician and finally HIV positive clients on ART who miss picking up a refill (every thirty days) are tracked.

##### Intervention fidelity

Fidelity is assessed at regular intervals through direct observation (visits by study PIs, investigators and overall research coordinator), monthly review of ICC process indicators and client satisfaction surveys administered to a convenience sample of 500 participants per site by staff not involved in delivery of services at the ICC.

#### Evaluation post-intervention assessment

A post-intervention assessment will be conducted in all 22 intervention and control sites approximately 24 months after the establishment of the ICCs (beginning in August 2016). RDS will be used to accrue samples of 1000 persons in each site using identical eligibility criteria as the baseline pre-intervention assessment. An additional module will be added to the survey to collect data on utilization and perceptions of the ICCs. Participants will undergo a blood draw and laboratory testing as in the pre-intervention assessment.

An important consideration is the selection of seeds for the post-intervention RDS. We will select either the same seeds, if possible (i.e., if the seeds are alive, residing in the same community and still regarded as peer leaders in the community) or seeds that are as similar as possible to the seeds used in the pre-intervention RDS (e.g., age, area of town they reside, marital status, sexual preference, drug of choice, etc.).

#### Data management systems

For the pre- and post-intervention assessments, we developed software in-house that references features of RDS coupon manager software and tracks recruitment and coupon numbers, links coupons of recruiters and recruits, tracks non-eligible referrals and determines reimbursements. The software also incorporates biometric data capture (fingerprint images) allowing storage of system-generated unique non-identifiable reference keys that are linked to study identification numbers. This biometric information is used to identify duplicate recruitments within and across sites in the pre- and post-intervention assessments. This information will also be used to identify participants who participated in both the pre- and post-intervention assessments and utilized an ICC.

Electronic surveys for the pre- and post-intervention interviews were developed using the Lime Survey Open Source Tool embedded with JAVA Scripts that includes logic checks, skip patterns and data constraints. The interview data is linked to the information captured in the in-house coupon manager software using the coupon ID. We utilize PHPMYADMIN with a secure password-protected encrypted database to store all data in a cloud. All data is exported from the individual sites via a Virtual Private Network tunnel to the central database maintained at YRGCARE in Chennai. When the local sites are connected to the internet, fingerprint–generated codes from all sites are pushed from the central server to the fingerprint database at each site. Storing this data centrally allows for the identification of duplicates in real-time within and across sites.

#### Laboratory testing and specimen storage

Laboratory specimens for the pre- and post-intervention surveys are collected on-site and processed and transported daily via courier to the central YRGCARE lab in Chennai for further testing and long-term storage. Only rapid HIV testing is performed on site where the data collection takes place. Additional CD4, HIV RNA, syphilis and HSV-2 testing are performed at YRGCARE. Indeterminate HIV results were resolved using Western Blot testing at YRGCARE. Remaining plasma and serum specimens are being stored at -70 °C at YRGCARE.

#### Regulatory oversight and participant safety

The trial is being conducted under regulatory review by institutional review boards at YRG CARE, Johns Hopkins School of Medicine, and Johns Hopkins Bloomberg School of Public Health. Additional trial oversight is provided by a data safety monitoring board (for the PWID stratum) and an advisory board (for the MSM stratum), both comprised of expert members external to the investigators’ organizations.

### Statistical power

We calculated power for comparing the primary outcome (HCT in prior 12 months) in intervention and control clusters at the post-intervention RDS survey [53]. We calculated the number of clusters needed assuming an outcome prevalence at the post-intervention RDS in control clusters of 10–40 %, a range of 350–1000 persons in each cluster for HIV-negative outcomes, an RDS design effect of 2, a two-sided α of 0.05, power = 0.80 and a within-stratum coefficient of variation ranging from 0.10 to 0.40. We incorporated the RDS design effect (relative to simple random sampling) by doubling the sample size required per cluster after calculating power. For the primary outcome, with 11 intervention and 11 control clusters, we will have 80 % power to detect an absolute difference of 12 % in the prevalence of the primary outcome in intervention and control clusters (e.g., 42 % and 30 %, respectively) with a within-stratum coefficient of variation of 0.25 and 1000 participants in each cluster.

Our trial was powered to be able to detect differences in the primary outcome, but we also calculated power for secondary outcomes. Power for secondary outcomes that include the full sample (e.g., proportion of PWID reporting sharing injection equipment in the prior 6 months) was similar to that for primary outcomes as the methods and the range of outcome prevalence in control clusters was within the range of what was considered for the primary outcome. Power was relatively insensitive to the within-cluster sample size so our ability to detect differences for outcomes where only HIV positive subjects are considered is similar to outcomes where the full RDS sample is used. For outcomes restricted to HIV-positive subjects, we assumed a sample size of 100–300 persons per cluster. Power remained insensitive to the cluster sample size down to a sample size of 100 per cluster. For example, with all other assumptions held constant and an RDS sample size of 150, if the baseline prevalence of the outcome is 30 %, we will be able to detect a difference of 14 percentage points (e.g., 30 % vs. 44 %). For outcomes with lower baseline prevalence, we would be able to detect a smaller difference (e.g., if 20 % of HIV infected patients are engaged in care in the control sites, we would have adequate power to detect a difference of 9 percentage points [20 % vs. 29 %]).

### Analysis plan

#### Community level

The primary analysis will be to compare the prevalence of community–level outcomes across intervention and control clusters, adjusting for pre-intervention prevalence levels. For a given outcome, we will first log-transform the 22 cluster-level proportions obtained from the post-intervention RDS. Then, via weighted least squares linear regression, these will be regressed on a dummy term for the control arm (vs. intervention), another for the MSM stratum (vs. PWID), and a term for the log-transformed cluster-level pre-intervention proportions. The exponentiated coefficient for the control arm term is thus the prevalence risk ratio (PRR), and (1-PRR) × 100 % is the percentage increase in service utilization associated with the intervention. The primary analyses will be conducted using the RDS-II weighted cluster-level proportions from both pre- and post-intervention RDS samples. These RDS-II weights, which account for personal network size (number of PWID or MSM seen in the past 30 days), will be calculated using the RDS Analyst Software Version 0.5 (http://hpmrg.org). For secondary outcomes that are continuous (e.g., community viral load) we will use a similar regression approach.

We will conduct several sensitivity analyses for the primary and all secondary outcomes First, we will repeat analyses using unweighted cluster-level proportions from both the pre-and post intervention RDS surveys. Second, we will consider adjustment for demographic covariates (age, sex, marital status and educational attainment) measured at the post-intervention RDS that are associated with the outcome and are differentially distributed across intervention and control clusters. We will consider adjustment for these factors if the p values for associations with the outcome and the intervention vs. control clusters are <0.05 and the OR is >2 or < 0.5. A two-stage approach will be used when adjusting for individual-level covariates: at the first stage, for a prevalence outcome, individual responses are modeled with a logistic regression model adjusting for all relevant covariates except the dummy term for control vs. intervention. In the second stage, observed and expected prevalence counts for each cluster are calculated, followed by *t*-test-like analyses of log-transformed ratios of observed to expected [53]. We will also consider an approach that models the difference in outcome prevalence between the pre- and post- intervention surveys using the same cluster-level comparison approach. We will also consider individual-level analyses using multi-level random effects regression approaches (Stata GLLAMMs program) to account for dependence of responses within clusters [54–56]. These models allow inclusion of fixed effects (e.g., intervention), random effects (e.g., clusters), adjustment for pre-intervention covariates at the individual and cluster level, and incorporation of scaled RDS weights as sampling weights. Finally, we will conduct descriptive analyses of the HIV care continuum, before and after the intervention phase of the study, in which completion of earlier steps are assumed to be necessary to complete later steps. Additionally, we will consider sensitivity analyses of outcomes in the HIV care continuum, where biologic markers such as HIV RNA and serum antiretroviral drug testing, are used to supplement self-reported data on access to care.

Several subgroup analyses are also planned. First, we will analyze all outcomes separately within each stratum (PWID and MSM). Using the combined sample of MSM and PWID sites, we will further compare all outcomes within subgroups defined by age, marital status, educational attainment, substance use (drug and alcohol use), and personal network size (number of persons in risk group [PWID or MSM] known and seen in the prior 30 days). Using only the PWID sites, we will also analyze subgroup differences by age, sex, marital status, educational attainment, substance use (including alcohol use), personal network size and region. In the MSM sites, we will also analyze subgroup differences by age, sexual identity, marital status, educational attainment, substance use (including alcohol use), personal network size and region. We also plan subgroup analyses by HIV serostatus and awareness of status for risk behaviors, HIV testing of spouses, substance use, stigma, and depression.

#### Network- and individual-levels

Network effects will be ascertained by comparing utilization of ICCs and services within ICCs across networks as defined by RDS. For example, we will examine utilization patterns across recruiters and recruits in the evaluation RDS. We will also ascertain whether utilization of ICCs varied by wave of RDS. Individual-level comparisons will draw on data from post-intervention RDS participants in the intervention clusters, in which extra questions will address participants’ use of ICC services. In addition, biometric data at the post-intervntion RDS will be linked with the biometric data from the ICCs to determine utilization. Individual analyses will use descriptive statistics and log-binomial regression to compare the level of each outcome (e.g., proportion accessing HCT in prior 12 months) by the main exposure of interest (visiting an ICC). We will adjust for individual-level confounders including demographic characteristics. Analyses will use multi-level random effects regression approaches to account for dependence of responses within personal networks [56]. In addition, using the biometric data to link persons between the pre-and post intervention RDS samples, we will conduct exploratory within-individual comparisons of the primary outcome and secondary outcomes. For example, restricting the sample to persons who participated in both the pre- and post intervention RDS samples and are eligible for the outcome, we will compare across control and intervention clusters the proportions of person who transition from not having the outcome to having it and vice versa.

### Dissemination

This trial represents a public-private partnership and collaboration between investigators at the Johns Hopkins University (Schools of Medicine and Public Health), investigators and research staff at the YR Gaitonde Centre for AIDS Research and Education (YRGCARE), the Targeted Intervention Division of the National AIDS Control Organization, India and several local State AIDS Control Organizations and local non-governmental organizations. Prior to and during the implementation of the trial, quarterly community meetings have been held to keep the community and relevant stakeholders informed on the conduct of the study. Meetings will continue past the end of the trial to inform the communities of the finding. A meeting will also be held with the representatives of the National AIDS control Organization to disseminate the findings. Once the trial is complete, depending on the findings, key stakeholders will decide on whether to adopt the ICC model as a whole or certain aspects of the model as the standard of care. This will require a consideration of the demonstrated effectiveness and cost. If they choose not to adopt the model, clients in sites with ICCs will be transferred back to the relevant government centers and NGOs for services (e.g., ART and OAT).

## Discussion

Dramatic progress has been made in the management and prevention of HIV since the first report in 1981. Yet, key challenges remain. First, implementation strategies need to be identified that can take demonstrated efficacious interventions at an individual level (e.g., ART, OAT) and improve their effectiveness in the real-world [37–40, 42]. Second, while ART roll-outs and expanded prevention services have led to overall declines in HIV prevalence across all settings [5], these declines have not been as apparent among key populations such as MSM and PWID [5, 57, 58]. In several countries where HIV epidemics are driven by drug use, HIV prevalence has at best stabilized if not increased over the past decade [5]. Resurgence in reports of both STIs and high-risk behavior have been noted globally among MSM [57]. These two populations are particularly difficult to target because a large majority of them remain hidden and no sampling frame exists making achieving a representative sample challenging. Our trial is unique in its utilization of RDS to evaluate the effectiveness of a community-level intervention in hard to reach populations.

Conversations about the end of AIDS and an AIDS-free generation have begun [59, 60]. However, for this goal to be realized, prevention, care and treatment services need to reach all populations at risk for HIV infection particularly those that are hardest-to-reach. There is a clear gap in access to services among MSM and PWID. Trials need to be designed to optimize utilization of services in these populations. We believe that this represents one of the first trials aimed at improving the HIV care continuum among MSM and PWID populations.

## Additional files

Additional file 1: MSM Baseline Survey. This survey was distributed (in local languages) to MSM study sites to gather baseline data on HIV status and risk behaviors. (PDF 4627 kb)
Additional file 2: PWID Baseline Survey. This survey was distributed (in local languages) to PWID study sites to gather baseline data on HIV status and risk behaviors. (PDF 1467 kb)

## Abbreviations

ART: Antiretroviral therapy
ATT: Anti-tuberculosis therapy
DOT: Directly observed therapy
FGD: Focus group discussions
HCT: HIV counseling and testing
ICC: Integrated care centers
IDI: In-depth interviews
MSM: Men who have sex with men
NGO: Non-governmental organizations
NSEP: Needle and syringe exchange programs
OAT: Opioid agonist therapy
PWID: People who inject drugs
RDS: Respondent-driven sampling
STI: Sexually transmitted infections
