# Supplementary material for: Design of the Indian NCA study (Indian national collaboration on AIDS): a cluster randomized trial to evaluate the effectiveness of integrated care centers to improve HIV outcomes among men who have sex with men and persons who inject drugs in India
Source: BMC Health Serv Res. 2016 Nov 14;16:652. doi: 10.1186/s12913-016-1905-5 (PMC5109686; doi:10.1186/s12913-016-1905-5)
Supplement: Additional file 1: — MSM Baseline Survey. This survey was distributed (in local languages) to MSM study sites to gather baseline data on HIV status and risk behaviors. (PDF 4627 kb) [file 12913_2016_1905_MOESM1_ESM.pdf]

The India Men's Health Initiative **Interviewer Initials ___ ___ ___**

Screening Form (SF) **Date ___ ___/___ ___/___ ___**

INTERVIEWER READ:
Hi, my name is ____________________ (Interviewer name). I would like to take this opportunity to ask you a few questions to find out a little bit about you. Some of the questions may be hard to answer. Please remember that all of your responses will be kept confidential and that I am not writing your name on this form. Please take as much time as you need. If you cannot, or do not wish to answer a particular question, tell me and I will go on to the next one. Remember, there are no right or wrong answers. Please answer them the best you can.

1. Is the participant a duplicate enrollment based on fingerprint?
- ☐ No 0 (→ Q2)
- ☐ Yes 1

INTERVIEWER INSTRUCTION: This will be determined at the front desk before the participant goes for screening. There will be a note on the checklist that the participant is a duplicate. The note will also include this persons original unique Study ID.

1a. Participant Study ID: ___ ___ ___ ___ ___ ___ [END SURVEY: Participant ineligible]

2. Does the participant have a valid RDS coupon?
- ☐ No 0
- ☐ Yes 1

INTERVIEWER INSTRUCTION: This will be determined at the front desk before the participant goes for screening. There will be a note on the checklist if the coupon is not valid.

3. What is your age in years?

___ ___ [Acceptable range: 10 – 98]
Years

4. How long have you lived where you are living now?

___ ___ [Acceptable range: 00 – 98]
Years

PROGRAMMER NOTE: Cannot be greater than age (Q3)
INTERVIEWER INSTRUCTION: Code 00 if less than 1 year.

5. Do you have any plans to move from here in the next two years?
- ☐ No 0
- ☐ Yes 1
- ☐ Don't Know 997
- ☐ Refused 998

6. In the past 12 months, have you had oral or anal sex with another man?
- ☐ No 0
- ☐ Yes 1

7. Have you ever been tested for HIV?
- ☐ No 0
- ☐ Yes 1
- ☐ Don't Know 997
- ☐ Refused 998

8. What is your biological sex?
   - ☐ Male — 1
   - ☐ Female — 2 (→ Q10)
   - ☐ Hijra/Transgender/Kinner — 3 (→ Q10)

9. How do you self-identify?
   - ☐ Panthi — 1
   - ☐ Kothi — 2
   - ☐ Double-decker — 3
   - ☐ Gay — 4
   - ☐ Bisexual — 5
   - ☐ MSM — 6
   - ☐ Other — 996

10. Are you currently employed?
   - ☐ No — 0
   - ☐ Yes — 1
   - ☐ Don't Know — 997
   - ☐ Refused — 998

INTERVIEWER INSTRUCTION: Questions 1, 2, 3, 6, and 8 should be used to determine eligibility.

NOT ELIGIBLE	ELIGIBLE
If any of the following is true:	*If all of the following is true:*
Q1 =1	Q1 =0
Q2 =0	Q2 =1
Q3 < 18 years of age	Q3 ≥ 18 years of age
Q6 = 0 (NO)	Q6 = 1 (YES)
Q8 = 2 or 3	Q8 = 1

PROGRAMMER NOTE: Use the criteria above to determine whether someone is eligible or not. A box should pop up on the screen to indicate whether a participant is eligible. This information needs to be linked back to the coupon manager program to determine reimbursement.

The India Men's Health Initiative

Baseline: Demographics (DG)

Participant ID ___ ___ ___ ___ ___
Date ___ ___/___ ___/___ ___
Interviewer Initials ___ ___ ___

GENERAL INTRODUCTION TO QUESTIONNAIRES

INTERVIEWER READ: I am going to ask you questions for about 45 minutes to 1 hour. I am going to ask questions about different behaviors and medical conditions you may have.

Some of the questions I ask may be uncomfortable to answer. You can choose not to answer any questions that you do not feel comfortable answering.

Please remember that all of the information you give us is confidential.

We ask that you answer the questions as honestly as you can.

If any of the questions are unclear, please stop me and I will try and make things clearer.

Remember, there are no right or wrong answers.

INTERVIEWER READ: I am going to start by asking you some questions about yourself and your living conditions.

1. What is your date of birth? ___ ___ / ___ ___ ___ / ___ ___ ___ ___
Day Month Year

[Range for Day: 1-31] [Range for Month: JAN-DEC] [Range for Year: 1912-2000]
INTERVIEWER INSTRUCTION: Use Date of Birth/Age recorded on Process Monitoring Form to fill this out. Enter 01/Jan YEAR if participant does not know the day/month.

2. Do you consider yourself to be?

- ☐ Panthi 1
- ☐ Kothi 2
- ☐ Double-decker 3
- ☐ Gay 4
- ☐ Bisexual 5
- ☐ MSM 6
- ☐ Satla Kothi 7
- ☐ Gupt Kothi 8
- ☐ Aqua Kothi 9
- ☐ Other 996

3. Which state does your family come from?

☐ Andaman and Nicobar	1
☐ Andhra Pradesh	2
☐ Arunachal Pradesh	3
☐ Assam	4
☐ Bihar	5
☐ Chandigarh	6
☐ Chhattisgarh	7
☐ Dadra & and Nagar Haveli	8
☐ Daman & Diu	9
☐ Delhi	10
☐ Goa	11
☐ Gujarat	12
☐ Haryana	13
☐ Himachal Pradesh	14
☐ Jammu and Kashmir	15
☐ Jharkhand	16
☐ Karnataka	17
☐ Kerala	18
☐ Lakshadweep	19
☐ Madhya Pradesh	20
☐ Maharashtra	21
☐ Manipur	22
☐ Meghalaya	23
☐ Mizoram	24
☐ Nagaland	25
☐ Orissa	26
☐ Puducherry	27
☐ Punjab	28
☐ Rajasthan	29
☐ Sikkim	30
☐ Tamil Nadu	31
☐ Tripura	32
☐ Uttar Pradesh	33
☐ Uttarakhand	34
☐ West Bengal	35
☐ Outside of India (Other)	996

INTERVIEWER INSTRUCTION: If mother and father are from different states, select state that father is from.

4. What is your religion?

☐ Hinduism	1
☐ Christianity	2
☐ Sikhism	3
☐ Buddhism	4
☐ Jainism	5
☐ Islam	6
☐ Atheist (Do not believe in god)	7
☐ Parsi	8
☐ Other	996

5. Are you now married, widowed, divorced, separated, never married, or living with a partner?
   - ☐ Married to female 1
   - ☐ Living with female partner, but not married 2
   - ☐ Living with male partner, but not married 3
   - ☐ Living with transgendered partner, but not married 4
   - ☐ In a long-term relationship, but not living with female partner 5
   - ☐ In a long-term relationship, but not living with male partner 6
   - ☐ In a long-term relationship, but not living with transgender partner 7
   - ☐ Widowed 8
   - ☐ Divorced 9
   - ☐ Separated from female partner but still married 10
   - ☐ Never married 11

INTERVIEWER INSTRUCTION: Participant must specify the gender of the partner.

6. What is the highest level of education you have completed?
   - ☐ No schooling 1
   - ☐ Primary school (1-5) 2
   - ☐ Secondary school (6-11) 3
   - ☐ High school / graduate (Complete 12th) 4
   - ☐ Vocational or trade school 5
   - ☐ College or university, not complete 6
   - ☐ College or university, complete 7
   - ☐ Post-graduate 8

7. What has been your usual earning pattern <u>during the last 12 months</u>? The answer should represent the majority of the last year, not just the most recent earning pattern.
   - ☐ Monthly wages 1
   - ☐ Weekly wages 2
   - ☐ Daily wages 3
   - ☐ Seasonally/intermittently employed 4
   - ☐ Temporarily laid off, sick leave 5
   - ☐ Unemployed, looking for work 6 (→ Q8)
   - ☐ Unemployed, not looking for work 7 (→ Q8)
   - ☐ Retired 8 (→ Q8)
   - ☐ Disabled, permanently or temporarily 9 (→ Q8)
   - ☐ Partner takes care of me / homemaker 10 (→ Q8)
   - ☐ Student 11 (→ Q8)

7a. What is your occupation?

☐	Private worker	1
☐	Government worker	2
☐	Own business	3
☐	Self-earning (coolie, auto driver, construction worker)	4
☐	Sex worker	5
☐	Work in massage parlour	6
☐	Housekeeping	7
☐	Catering	8
☐	Beautician	9
☐	Pimp / Broker	10
☐	Other	996

8. During the past 12 months, what was your average personal monthly income?

___ ___ ___ ___ ___ ___ rupees per month

9. During the past 12 months, what was the average income of your household?

___ ___ ___ ___ ___ ___ rupees per month

PROGRAMMER NOTE: Answer to Q9 cannot be less than answer to Q8.

INTERVIEWER READ: We now have a few questions about where you are living.

10. In what type of place do you currently live?

☐	Own house / condominium / apartment / flat	1
☐	Rent house / condominium / apartment / flat	2
☐	Rent room in someone else's house	3
☐	Staying with family member(s)/parents	4
☐	Stay with friend(s)/others	5
☐	Staff quarters	6
☐	Hostel/Dormitory	7
☐	On the street, in a park, in a bus station, park etc.	8
☐	With a DERA	9
☐	In a slum	10

10a. What is your current pin/zip code? ___ ___ ___ ___ ___ ___

Range: 100000-999997

INTERVIEWER INSTRUCTION:

- Code 999997 for Don't Know
- Code 999996 if Living on the Street

PROGRAMMER NOTE: If Q10 = 8, then code this question as not applicable "999996"

11. How long have you been living where you currently live?

☐	Less than 1 month	1
☐	More than 1 month to 3 months	2
☐	More than 3 months to 6 months	3
☐	More than 6 months to 1 year	4
☐	More than 1 year	5

12. How many individuals live in your household, not counting yourself? By household, I mean children or adults (not including yourself) who sleep in the household at least two nights or more every week.

___ ___ ___
[Acceptable range: 0-30, 996]

INTERVIEWER INSTRUCTION: If living in a hostel, participant can estimate number of person or Code 996 for Not Applicable

PROGRAMMER NOTE: If Q12 =0, skip to Q13

12a. Who currently lives with you? (**SELECT ALL THAT APPLY**)

	Yes	No
12a1. Spouse/partner	1	0
12a2. Your children / partner's children	1	0
12a3. Parent(s) and or grandparent(s)	1	0
12a4. Siblings	1	0
12a5. Other relative(s)	1	0
12a6. Friend(s)	1	0
12a7. Other members of DERA	1	0
12a8. Other persons living in hostel	1	0
12a9. Renter(s)	1	0
12a10. Sex worker(s)	1	0
12a11. Other	1	0

PROGRAMMER NOTE: Q12a1-12a11 should all be on one page
- Number of options checked should not be greater than the answer to Q12.

INTERVIEWER INSTRUCTION: Please read all choices to participant.

13. How many children do you have? ___ ___ [Acceptable range: 0-10]

14. In the last 12 months, how often did you run out of money for basic necessities like housing or food?
- ☐ Never 1
- ☐ Once or twice 2
- ☐ Monthly 3
- ☐ Weekly 4
- ☐ Daily 5
- ☐ Don't know 997

15. In the last 12 months, how many months of the year did you stay away from your home (for work/school etc. so that you could not sleep at home)?
- ☐ None 0
- ☐ Less than 1 month 1
- ☐ More than 1 month to 3 months 2
- ☐ More than 3 months to 6 months 3
- ☐ More than 6 months to1 year 4
- ☐ Don't Know 997

16. In the last 12 months, what is the furthest distance that you traveled (by motorbike, car, train or plane)?

___ ___ ___ ___ km
[Range: 0-9997]

INTERVIEWER INSTRUCTION: Code 9996 if more than 9995 km. Code 9997 for Don't Know;

PROGRAMMER NOTE: If Q16=0 then automatically code 17j as 1

17. In the last 12 months, did you travel for any of the following reasons? (**SELECT ALL THAT APPLY**)

	Yes	No
17a. Work	1	0
17b. School	1	0
17c. Visit family home town / attend family function	1	0
17d. Holiday / tour / excursion	1	0
17e. For sexual partners	1	0
17f. For drugs	1	0
17g. To meet friends	1	0
17h. To attend a cultural program or temple festival	1	0
17i. My home is in another state	1	0
17j. I did not travel in the last 12 months	1	0
17k. Other	1	0

PROGRAMMER NOTE: Q17a-17k should all be on one page.
INTERVIEWER INSTRUCTION: Please read all choices to participant.

18. What is your sexual orientation?

- ☐ Heterosexual/straight 1
- ☐ Homosexual/Gay 2
- ☐ Bisexual 3
- ☐ Other 996

The India Men's Health Initiative

Baseline Network Questions (NW)

Participant ID ___ ___ ___ ___ ___
Date ___ ___/___ ___/___ ___
Interviewer Initials ___ ___ ___

INTERVIEWER READ: The next few questions are about people you may know who are like you. If you don't know the exact number for some of the questions I ask, please give your best guess.

1. Which of the following describes how you know the person who gave you the coupon that you brought to this study? (**SELECT ALL THAT APPLY**)

	Yes	No
1a. A friend	1	0
1b. A sex partner or boyfriend	1	0
1c. A client	1	0
1d. A relative or family member	1	0
1e. A co-worker	1	0
1f. An acquaintance (a person you know, but do not consider a friend)	1	0
1g. You don't know the person / just met them (a stranger)	1	0
1h. A sex worker	1	0

INTERVIEWER INSTRUCTION: Please read all choices to participant.
PROGRAMMER NOTE: Questions 1a-1h should all be on one page.

INTERVIEWER READ: When I use the term MSM, I am referring to all men who you know that have sex with other men. This includes panthis, kothis, DDs, gays, and bisexuals.

2. How many people in [**project city**] do you personally know who are MSM?
___ ___ ___ [Acceptable range: 0- 200]
INTERVIEWER INSTRUCTION: If participant says that they don't know, PROBE to come up with an answer.
PROGRAMMER NOTE: If 0, END SURVEY.

- Pre-populate Project City based on survey location.

3. Of the [**Q2 answer**] people who live in **[project city],** who you personally know are MSM, how many have you seen at least once in the past 30 days?
___ ___ ___ [Acceptable range: 0- 200]
INTERVIEWER INSTRUCTION: If participant says that they don't know, PROBE to come up with an answer.
PROGRAMMER NOTE: If 0, END SURVEY

- Q3 cannot be greater than Q2.
- Pre-populate Q2 answer in question.
- Prepopulate Project City based on survey location.

4. Of the [**Q3 answer**] MSM you have seen in the past 30 days, how many are kothi?
___ ___ ___ [Acceptable range: 0- 200, 997, 998]
INTERVIEWER INSTRUCTION: Code 997 for Don't Know; 998 for Refused.
PROGRAMMER INSTRUCTION: Q4 cannot be greater than Q3.

- Pre-populate Q3 answer in question

5. Of the [**Q3 answer**] MSM you have seen in the past 30 days, how many are panthi?
___ ___ ___ [Acceptable range: 0- 200, 997, 998]
INTERVIEWER INSTRUCTION: Code 997 for Don't Know; 998 for Refused.
PROGRAMMER NOTE: Q5 cannot be greater than Q3.

- Pre-populate Q3 answer in question

6. Of the [**Q3 answer**] MSM you have seen in the past 30 days, how many are DD?
___ ___ ___ [Acceptable range: 0- 200, 997, 998]
INTERVIEWER INSTRUCTION: Code 997 for Don't Know; 998 for Refused.
PROGRAMMER NOTE: Q6 cannot be greater than Q3.
- Pre-populate Q3 answer in question

7. Of the [**Q3 answer**] MSM you have seen in the past 30 days, how many are bisexual?
___ ___ ___ [Acceptable range: 0- 200, 997, 998]
INTERVIEWER INSTRUCTION: Code 997 for Don't Know; 998 for Refused.
PROGRAMMER NOTE: Q7 cannot be greater than Q3.
- Pre-populate Q3 answer in question

8. Of the [**Q3 answer**] MSM you have seen in the past 30 days, how many are gay?
___ ___ ___ [Acceptable range: 0- 200, 997, 998]
INTERVIEWER INSTRUCTION: Code 997 for Don't Know; 998 for Refused.
PROGRAMMER NOTE: Q8 cannot be greater than Q3.
- Pre-populate Q3 answer in question

9. Of the [**Q3 answer**] MSM that you have seen in the past 30 days, how many are from [**Neighborhood where study is being conducted]**?
___ ___ ___ [Acceptable range: 0- 200, 997, 998]
INTERVIEWER INSTRUCTION: Neighborhood is the area of the city where the study clinic is.
Code 997 for Don't Know; 998 for Refused.
PROGRAMMER NOTE: Q9 cannot be greater than Q3.
- Pre-populate Q3 answer in question.

The India Men's Health Initiative

Baseline: HIV testing & Medication History (HIV)

Participant ID ___ ___ ___ ___ ___
Date ___ ___/___ ___/___ ___
Interviewer Initials ___ ___ ___

INTERVIEWER READ: The next few questions are about HIV and your experiences with testing and treatment for HIV.

HIV TESTING EXPERIENCE

1. Have you ever been tested for HIV?
   - ☐ No 0
   - ☐ Yes 1 (→ Q3)

2. Have you not been tested…**(SELECT ALL THAT APPLY)**	**No**	**Yes**
2a. because you think you are at low risk for HIV infection?	0	1
2b. because you were afraid of finding out that you had HIV?	0	1
2c. because you were worried your name would be reported to the government if you tested positive?	0	1
2d. because you were afraid of someone finding out about the test result?	0	1
2e. because you were afraid of losing your job, insurance, or housing if you tested positive?	0	1
2f. because you didn't have the money or the insurance to pay for the test?	0	1
2g. because you didn't have time?	0	1
2h. because you didn't know where to go to get tested?	0	1
2i. because you couldn't get transportation to a testing place?	0	1
2j. because you don't like needles?	0	1
2k. because you were afraid your relatives or friends would see you if you went to a public place like a government testing center	0	1
2l. because you heard about someone being treated badly when they went for an HIV test	0	1
2m. Refused to answer	0	1

INTERVIEWER INSTRUCTION: Please read all options 2a-2l to participant.

PROGRAMMER NOTE: Q2a-2m should be on one page

- If option 'm' is selected, skip to Q9.
- If any of 'a-l' is selected at the same time as 'm' then a pop-up box should appear 'You should not select DON'T KNOW/REFUSED when you have selected any other options. Please check answers.'

2a1. What is the primary reason you have never been tested for HIV infection?

- ☐ Because you think you are at low risk for HIV infection 1
- ☐ Because you were afraid of finding out that you had HIV 2
- ☐ Because you were worried your name would be reported to the government if you tested positive 3
- ☐ Because you were afraid of someone finding out the test result 4
- ☐ Because you were afraid of losing your job, insurance, or housing if you tested positive 5
- ☐ Because you didn't have the money or insurance to pay for the test 6
- ☐ Because you didn't have time 7
- ☐ Because you didn't know where to get tested 8
- ☐ Because you couldn't get transportation to a testing place 9
- ☐ Because you don't like needles 10
- ☐ Because you were afraid relatives or friends would see you if you went to a public place like a government testing center 11
- ☐ Because you heard about someone being treated badly when they went for an HIV test 12

PROGRAMMER NOTE: Only an option selected as 1 'Yes' in Q2 can be selected here

- SKIP to Q9

INTERVIEWER READ: I want to ask you some questions about the last time you were tested for HIV.

3. Do you remember when you were last tested for HIV?
   - ☐ Yes 1 (→3a)
   - ☐ No 2 (→ 3b)

3a. When were you last tested for HIV? ___ ___ / ___ ___ ___ / ___ ___ ___ ___
Day Month Year

[Range for Day: 01-31] [Range for Month: JAN-DEC] [Range for Year: date of birth - present date]

PROGRAMMER NOTE: Skip to Q4.

3b. What is the best estimate of when you were last tested for HIV?

- ☐ Within the last month 1
- ☐ More than 1 month to 6 months ago 2
- ☐ More than 6 months to 1 year (12 months) ago 3
- ☐ More than 1 year to 2 years ago 4
- ☐ More than 2 years ago to 4 years ago 5
- ☐ More than 4 years ago 6

INTERVIEWER INSTRUCTION: Ask question and let participant respond openly. Then fit answer into the best option. If participant does not remember, probe to help him come up with an answer.

4. The last time you were tested for HIV, why did you get tested for HIV?

☐	I wanted to know my status	1
☐	Condom tore / did not use a condom and I was worried	2
☐	Because I engage in sex work	3
☐	Because I shared needles/syringes with someone	4
☐	Symptoms	5
☐	As part of a research study	6
☐	An outreach worker took me to have a test	7
☐	My regular partner is unwell / took me to get tested / tested positive	8
☐	Someone I know tested HIV positive	9
☐	A friend/network partner of mine was getting tested for HIV	10
☐	A family member of mine was getting tested for HIV	11
☐	My doctor suggested I get tested	12
☐	I was forced to do a test	13
☐	I was diagnosed with tuberculosis	14
☐	My wife is pregnant	15
☐	Because I am getting married	16
☐	As part of a targeted intervention (TI)	17
☐	I get tested regularly	18
☐	Other	996
☐	Don't know	997
☐	Refused	998

INTERVIEWER INSTRUCTION: Ask question and let participant respond openly. Then fit answer into the best option.
- If participant selects more than one reason, ask them to select main reason.

5. Where were you last tested for HIV? ___ ___ ___
[Range: 01-20, 996, 997, 998]

INTERVIEWER INSTRUCTION: Use **Card HIVSITE**
- Code 996 for other; 997 for Don't Know; 998 for Refused

5a. What was this location?

☐	Government voluntary counseling & testing center (ICTC)	1
☐	Private/NGO voluntary counseling & testing center	2
☐	Jail / prison	3
☐	Government hospital	4
☐	Private hospital	5
☐	Private laboratory (stand-alone lab)	6
☐	Syringe exchange program	7
☐	OST/methadone center (drug treatment)	8
☐	Donating blood or plasma	9
☐	Family planning center	10
☐	As part of a research study	11
☐	Government ART center	12
☐	Other	996
☐	Don't know	997
☐	Refused	998

INTERVIEWER INSTRUCTION: Use **Card HIV5**.

6. Did the last HIV test you took use a swab from your mouth, blood from your finger, or blood from your arm?

☐ Swab from mouth	1
☐ Blood from finger (finger prick)	2
☐ Blood from arm (needle stick)	3
☐ Other	996
☐ Don't know	997
☐ Refused	998

7. The last time you were tested for HIV, how quickly did you receive the results of the test?

☐ Did not receive result	0
☐ Within the hour	1 (→ Q8)
☐ More than an hour, but within the same day	2 (→ Q8)
☐ The following day	3 (→ Q8)
☐ More than a day, but within the week	4 (→ Q8)
☐ More than a week later	5 (→ Q8)
☐ Don't know	997 (→ Q8)
☐ Refused	998 (→ Q8)

7a. Think about the last time you didn't get your HIV test result. What was the main reason you didn't get your result?

☐ Too early to get the result	1
☐ Thought site would contact you	2
☐ Afraid of getting result	3
☐ Too busy to get the result	4
☐ Forgot to get result	5
☐ Didn't care about result/didn't want to know	6
☐ Jail-related (incarcerated/released before getting result)	7
☐ Inconvenient (location/hours/time etc.)	8
☐ Lost appointment card, paperwork, ID number	9
☐ I already knew I was HIV-infected	10
☐ Worried about someone seeing me there or identifying me	11
☐ I went out of town (e.g., went to home town)	12
☐ Other	996
☐ Don't know	997
☐ Refused	998

INTERVIEWER INSTRUCTION: Ask question and let participant respond openly. Then fit answer into the best option.

PROGRAMMER NOTE: Skip to Q9.

8. What were the results of your last HIV test?

☐ Negative	0
☐ Positive	1 (→ Q11)
☐ Indeterminate	2
☐ Don't remember	3

9. Have you ever been told that you had HIV?

☐ No	0 (→ Q55)
☐ Yes	1

9a. Where were you told you have HIV?

- ☐ Government voluntary counseling & testing center (ICTC) 1
- ☐ Private/NGO voluntary counseling & testing center 2
- ☐ Jail / prison 3
- ☐ Government hospital 4
- ☐ Private hospital 5
- ☐ Private laboratory (stand-alone lab) 6
- ☐ Syringe exchange program 7
- ☐ OST/methadone center (drug treatment) 8
- ☐ Donating blood or plasma 9
- ☐ Family planning center 10
- ☐ As part of a research study 11
- ☐ Government ART center 12
- ☐ Other 996
- ☐ Don't know 997
- ☐ Refused 998

INTERVIEWER INSTRUCTION: Use **Card HIV5**.

10. When were you told you had HIV?

- ☐ Within the last month 1
- ☐ More than 1 month to 6 months ago 2
- ☐ More than 6 months to 1 year (12 months) ago 3
- ☐ More than 1 year to 2 years ago 4
- ☐ More than 2 years ago to 4 years ago 5
- ☐ More than 4 years ago 6

INTERVIEWER INSTRUCTION: Ask question and let participant respond openly. Then fit their answer into the best option.

INTERVIEWER READ: You have told us that you are HIV positive. I would like to ask you some questions about when you found out you were HIV positive and what happened after that diagnosis.

11. Have you ever told anyone in your personal life that you are HIV positive (not including a health professional)? By this, I mean have you told a friend, a family member, a sexual partner or someone else?

- ☐ No 0 (→ Q12)
- ☐ Yes 1
- ☐ Refused 998 (→ Q12)

11a. Who have you told that you are HIV positive? (**SELECT ALL THAT APPLY**)

	**Yes**	**No**
11a1. Spouse / primary female sexual partner	1	0 (→ Q12)
11a2. Primary male sexual partner	1	0 (→ Q12)
11a3. Casual sex partner	1	0
11a4. MSM friends	1	0
11a5. Friends who are not MSM	1	0
11a6. Co-worker(s)	1	0
11a7. Family member	1	0
11a8. Counselor - NGO/CBO worker	1	0
11a9. Doctor, nurse or health care professional	1	0

PROGRAMMER NOTE: Q11a1-Q11a9 should all be on one page

- If either 11a1 or 11a2 is equal to 0 (No), go to Q12. Otherwise skip to Q13.

INTERVIEWER INSTRUCTION: Please read all choices to participant.

12. Why have you not told your spouse/primary sexual partner that you are HIV positive?

☐ Fear of rejection/abandonment / wife leaving you	1
☐ Fear of psychological abuse	2
☐ Fear of physical abuse	3
☐ Fear of withdrawal of financial support	4
☐ Fear of not being able to be with children	5
☐ Fear of being blamed for being unfaithful	6
☐ HIV status is a personal issue; want to keep it a secret	7
☐ Did not think it was necessary or important	8
☐ Unable to accept / digest news	9
☐ Fear of stigma / discrimination	10
☐ Fear of losing sexual relationship with spouse / partner	11
☐ I do not have a spouse/primary sexual partner	12
☐ Other	996
☐ Don't know	997
☐ Refused	998

INTERVIEWER INSTRUCTION: Ask question and let participant respond openly. Then fit answer into the best option.

- If participant selects more than one reason, ask them to select main reason.

INTERVIEWER READ: I am now going to ask you about any medical care and treatment you have received for HIV.

13. When you found out you were HIV positive, did you get any help in getting HIV-related medical care? By help we mean help with an appointment, where to go, transportation and paperwork?

☐ No	0 (→ Q16)
☐ Yes	1
☐ Refused	998 (→ Q16)

14. Who gave you this help?

☐ Counselor at a government center	1
☐ Counselor/worker at a CBO or NGO	2
☐ Doctor, nurse, or health care professional at a private hospital or clinic	3
☐ Doctor, nurse or health care professional at an NGO or CBO	4
☐ Government doctor, nurse or health care professional	5
☐ Other	996
☐ Refused	998

INTERVIEWER INSTRUCTION: If participant chooses more than one answer, ask him to choose the person who was most helpful.

15. How long after you received your HIV positive result did you receive this help?

☐ Within the first 2 weeks of getting result	1
☐ More than 2 weeks to 1 month after getting the result	2
☐ More than 1 month to 3 months after getting the result	3
☐ More than 3 months to 6 months after getting the result	4
☐ More than 6 months to 1 year after getting the result	5
☐ More than 1 year after getting the result	6
☐ Don't know	997
☐ Refused	998

INTERVIEWER INSTRUCTION: Ask question and let participant respond openly. Then fit answer into the best option.

16. Did you receive any of the following counseling after you found out you were HIV positive? (**SELECT ALL THAT APPLY**)

	**Yes**	**No**
16a. Individual counseling on HIV risk reduction to prevent transmission of HIV to others	1	0
16b. Information and counseling on medical/clinical care and treatment options available to help fight HIV and keep healthy	1	0
16c. Counseling to address anxiety and depression	1	0
16d. Counseling on healthy living (e.g., nutrition and exercise)	1	0
16e. Couples counseling	1	0
16f. Information about having children	1	0
16g. Family counseling	1	0
16h. Refused to answer	1	0

INTERVIEWER INSTRUCTION: Please read all choices to participant.
PROGRAMMER NOTE: Q16a-16h should all be on one page.
If option '16h' is selected, all previous responses '16a-g' should be set to missing.

17. Have you ever been to see a doctor for the management of your HIV?
- ☐ No 0
- ☐ Yes 1 (→ Q19)

18. Why have you not been to see a doctor about the management of HIV?
- ☐ I am not thinking about HIV medical care at this time 1
- ☐ HIV medical care is important to me but I am not ready to go to a doctor yet 2
- ☐ HIV medical care is important to me but I have not tried to find a doctor or clinic yet 3
- ☐ I have found a doctor but have not made an appointment yet / have not gone yet 4
- ☐ I have tried to obtain HIV medical care but I have not been successful 5
- ☐ I do not know where to go for HIV medical care 6
- ☐ I do not have the money for HIV medical care 7
- ☐ I do not want to go to the government center for HIV medical care 8
- ☐ There is no cure for HIV and so no point to seeing a doctor 9
- ☐ There is no treatment for HIV and so no point to going to see a doctor 10
- ☐ HIV medicines have a lot of side effects so I am not interested 11
- ☐ My friend/sexual partner was treated badly when they went for HIV medical care 12
- ☐ Other 996
- ☐ Don't know 997
- ☐ Refused 998

INTERVIEWER INSTRUCTION: Ask question and let participant respond openly. Then fit answer into the best option.
- If participant selects more than one reason, ask them to select main reason.

PROGRAMMER NOTE: Skip to Q26.

[Type]	a. Do you see a [type] for management of your HIV?		b. How often do you see this [type]?					c. When was the last time you saw this [type]?
	Yes	No	Once a month	Once in 3 months	Once in 6 months	Once per year	Less than once per year	Day/Month/Year
19. private medical doctor (in stand-alone clinic)	1	0 [20]	1	2	3	4	5	__ __ / _ _ _ /__ __ __ __
20. private medical doctor (in hospital)	1	0 [21]	1	2	3	4	5	__ __ / _ _ _ /__ __ __ __
21. government hospital doctor	1	0 [22]	1	2	3	4	5	__ __ / _ _ _ /__ __ __ __
22. NGO doctor	1	0 [23]	1	2	3	4	5	__ __ / _ _ _ /__ __ __ __
23. alternative / non-allopathic doctor (ayurveda/siddha)	1	0 [24]	1	2	3	4	5	__ __ / _ _ _ /__ __ __ __
24. pharmacist /chemist	1	0 [25]	1	2	3	4	5	__ __ / _ _ _ /__ __ __ __
25. any other type of doctor	1	0 [26]	1	2	3	4	5	__ __ / _ _ _ /__ __ __ __

[Range for Day: 01-31] [Range for Month: JAN-DEC] [Range for Year: date of birth-present date]

INTERVIEWER INSTRUCTION for 19-25a: Doctor includes Doctor, Nurse or Health Care Professional.

INTERVIEWER INSTRUCTION FOR 19-25b: Use **Card HIV19**

PROGRAMMER NOTE: Include interviewer instruction for 19-25a on each page for QA (19-25)

- Include interviewer instruction for 19-25b on each page for QB (19-25)
- [x] means to skip the following a-c questions and go onto to next numbered question.
- If Q17 is = 1 (Yes) then at least one of Q19a-Q25a has to be equal to 1 (Yes)

26. Have you ever had a CD4 cell count?
- ☐ No 0 (→ Q30)
- ☐ Yes 1
- ☐ Don't Know 997 (→ Q30)

INTERVIEWER INSTRUCTION: Ask for everyone, even those not in care.

27. When did you receive a CD4 cell count in relation to your first positive HIV test?
- ☐ Within the first 2 weeks of getting the result 1
- ☐ More than 2 weeks to 1 month after getting the result 2
- ☐ More than 1 month to 3 months after getting the result 3
- ☐ More than 3 months to 6 months after getting the result 4
- ☐ More than 6 months to 1 year after getting the result 5
- ☐ More than 1 year after getting the result 6
- ☐ Don't know 997

INTERVIEWER INSTRUCTION: Ask question and let participant respond openly. Then fit answer into the best option.

28. When is the last time you received a CD4 cell count?
- ☐ Within the last month 1
- ☐ More than 1 month to 6 months ago 2
- ☐ More than 6 months to 1 year (12 months) ago 3
- ☐ More than 1 year to 2 years ago 4
- ☐ More than 2 years ago to 4 years ago 5
- ☐ More than 4 years ago 6
- ☐ Don't know 997

INTERVIEWER INSTRUCTION: Ask question and let participant respond openly. Then fit answer into the best option.

29. What was your most recent CD4 cell count? ___ ___ ___ ___ cells/mm^3 [Acceptable range: 0-1500, 9997]
INTERVIEWER INSTRUCTION: Code 9997 for Don't Know.

ANTIRETROVIRAL THERAPY USE

30. Have you ever taken antiretroviral medications/HIV tablets/ "dabba marandhe" for HIV?
- ☐ No 0
- ☐ Yes 1 (→ Q31)

30a. Why have you not taken antiretroviral medications/medicines to treat HIV?
- ☐ I do not need HIV medicines/My CD4 count is high 1
- ☐ I do not know where to go to get ART 2
- ☐ The ART clinic is too far for me 3
- ☐ There was no ART available at the place where I got tested 4
- ☐ ART medications are harmful 5
- ☐ ART medicines do not work 6
- ☐ ART medicines have bad side effects 7
- ☐ I have heard stories about people being treated badly at ART clinics 8
- ☐ I am healthy. I do not need ART. 9
- ☐ I am too busy 10
- ☐ Because I am drinking 11
- ☐ Because I do not want to take the medications around my friend/partner 12
- ☐ Did now know there were medicines for HIV 13
- ☐ Other 996

PROGRAMMER INSTRUCTION: Skip to Q53
INTERVIEWER INSTRUCTION: Ask question and let participant respond openly. Then fit answer into the best option.
- If participant selects more than one reason, ask them to select main reason.

31. When did you first start taking antiretroviral medication for HIV?
(Estimate okay): ___ ___ / ___ ___ ___ / ___ ___ ___ ___
Day Month Year

[Range for Day: 01-31] [Range for Month: JAN-DEC] [Range for Year: 1900, 1985-present date]
PROGRAMMER NOTE: CODE 01 JAN 1900 for Don't Know (when interviewer leaves answer blank).
- If answer is 01 JAN 1900, remove logic checks

INTERVIEWER INSTRUCTION: Leave as 01 JAN 1900 for Don't Know; if participant knows YEAR only, code 01 JAN YEAR

32. Who prescribed these antiretroviral medications for HIV to you? (**SELECT ALL THAT APPLY**)

	Yes	No
32a. Private medical doctor (in stand-alone clinic)	1	0
32b. Private medical doctor (in hospital)	1	0
32c. Government hospital doctor	1	0
32d. NGO doctor	1	0
32e. Alternative / non-allopathic doctor (ayurveda/ siddha/ quack)	1	0
32f. Pharmacist /chemist	1	0
32g. Other	1	0
32h. Don't Know	1	0
32i. Refused	1	0

INTERVIEWER INSTRUCTION: Use **Card HIV32**.
PROGRAMMER NOTE: Q32a-32i should all be on one page
- If any of 'a-g' is selected at the same time as 'h' or 'I' then a pop-up box should appear 'You should not select DON'T KNOW/REFUSED when you have selected any other options. Please check answers.'

33. In the past 30 days, did you take any HIV medications?
- ☐ No 0
- ☐ Yes 1

INTERVIEWER READ: I would like to ask about HIV medications you may have taken. Please tell me all of the medications that you have EVER taken.

	**a. Have you ever taken [medication]?**		**b. Have you taken [medication] in the last 30 days?**	
	**No**	**Yes**	**No**	**Yes**
34. Stavudine+ Lamivudine+ Nevirapine (Triomune 30/40, Stavex-LN)	0 [35]	1	0	1
35. Stavudine + Lamivudine (Lamivir-s 30/40, Stavex L)	0 [36]	1	0	1
36. Zidovudine + Lamivudine + Nevirapine (Duovir-N, Zidovex LN)	0 [37]	1	0	1
37. Zidovudine + Lamivudine (Duovir, Zidovex L)	0 [38]	1	0	1
38. Zidovudine + Lamivudine + Efavirenz (Duovir – E kit, Zidovex LE)	0 [39]	1	0	1
39. Efavirenz (EFCURE 600, Efavir 600, Effereven 600, Viranz 600)	0 [40]	1	0	1
40. Tenofovir + Emtricitabine (Tenvir EM, Fostravir EM)	0 [41]	1	0	1
41. Tenofovir + Lamivudine (Tenvir-L, Tavin L)	0 [42]	1	0	1
42. Tenofovir + Emtricitabine + Efavirenz (Viraday, Tofoday, Fostavir 3, Vonavir)	0 [43]	1	0	1
43. Tenofovir (Tenvir, Tavin)	0 [44]	1	0	1
44. Atazanavir (Atazor, Atavir)	0 [45]	1	0	1
45. Ritonavir + Lopinavir (Lopimune, Aluvia, HIVUS LR, Emletra, Kaletra)	0 [46]	1	0	1
46. Ritonavir (Ritomune, Empetus)	0 [47]	1	0	1
47. any other antiretroviral medications for the treatment of HIV	0 [48]	1	0	1
48. any other antiretroviral medication that you don't know the name of (1)	0 [50]	1	0	1
49. any other antiretroviral medication that you don't know the name of (2)	0 [50]	1	0	1

INTERVIEWER INSTRUCTION for Q34-Q46: Use **Cards HIV34-46.**
PROGRAMMER NOTE: For each Question, the interviewer instruction should say to use the corresponding card. For example, For 34a and 34b, the interviewer instruction should read: Use **Card HIV34**
- [x] means to skip b and go onto to next numbered question.

INTERVIEWER INSTRUCTION for Q47: Use Card 47. If the drug is not listed on **Cards HIV34-46** and you do not know which category it fits into, code "YES." Do not include homeopathic drugs in this question.

INTERVIEWER INSTRUCTION for Q48-Q49: Code YES if the participant reports taking ART but says they do not know the name of the medication. Do not include homeopathic drugs in this question.

**Medication name**	**c. How many tablets do you take at one time?**	**d. How many times per day do you take that dose?**	**e. How are you HIV tablets packaged?**
48. Don't know (1)	______ [Range: 1-6, 997]	______ [Range: 1-3, 997]	☐ From a bottle/box 1<br>☐ From a strip 2<br>☐ From a bottle/box and strip 3<br>☐ Don't Know 997<br>☐ Refused 998
49. Don't know (2)	______ [Range: 1-6, 997]	______ [Range: 1-3, 997]	☐ From a bottle/box 1<br>☐ From a strip 2<br>☐ From a bottle/box and strip 3<br>☐ Don't Know 997<br>☐ Refused 998

PROGRAMMER NOTE: At least 1 of 34-49a has to = 1.
If Q33=0, go to Q50.
If Q33 =1 go to Q52.

50. When did you stop taking HIV medications?
(Estimate okay): ___ ___ / ___ ___ ___ / ___ ___ ___ ___
Day Month Year

[Range for Day: 01-31] [Range for Month: JAN-DEC] [Range for Year: 1900, 1985-present date]
PROGRAMMER NOTE: CODE 01 JAN 1900 for Don't Know (when interviewer leaves answer blank).

- Date for Q50 cannot be less than Q31 (Unless answer is 01 JAN 1900)

INTERVIEWER INSTRUCTION: Leave as 01 JAN 1900 for Don't Know; if participant knows YEAR only, code 01 JAN YEAR

51. Why did you stop taking HIV medications? (**SELECT ALL THAT APPLY**)

	**Yes**	**No**
51a. My HIV is cured	1	0
51b. The medications made me sick (side effects)	1	0
51c. The doctor told me to stop	1	0
51d. I was too busy doing other things to take medicine	1	0
51e. Medicines were too difficult to take	1	0
51f. Other illness/health problems got in the way	1	0
51g. Ran out of money to buy medicines	1	0
51h. The medicines were not working	1	0
51i. Didn't like going to the government center to collect ART	1	0
51j. The doctors treated me badly	1	0
51k. Someone else at the ART center treated me badly	1	0
51l. I feel better	1	0
51m. Don't Know	1	0
51n. Refused	1	0

INTERVIEWER INSTRUCTION for Q51: Please read all choices a-l to participant. Use **Card HIV51**.
PROGRAMMER NOTE: Q51a-51n should all be on one page

- If any of 'a-l' is selected at the same time as 'm' or 'n' then a pop-up box should appear 'You should not select DON'T KNOW/REFUSED when you have selected any other options. Please check answers.'
- SKIP to Q53

52. Think about the last 30 days and how many times you should have taken your HIV medications. Please point on the line showing the number that is your best guess about how much medicine you have taken in the past 30 days?

0% means you have taken no HIV medicine, 50% means you have taken half of the medicine you were supposed to take, and 100% means you have taken every single dose of the HIV medicine you were supposed to take.

0% 10% 20% 30% 40% 50% 60% 70% 80% 90% 100%

___ ___ ___ [Range: 0-100, 997, 998]
INTERVIEWER INSTRUCTION: Use **Card HIV52.**
Code 997 for Don't Know, 998 for Refused.
PROGRAMMER NOTE: Just provide a space to enter the number 0-100; No need to draw out the scale.

53. Have you ever taken any homeopathic/ayurvedic/herbal medications for HIV?

- ☐ No 0
- ☐ Yes 1
- ☐ Don't Know 997
- ☐ Refused 998

54. Have you ever taken TMP-SMX [or TMP-SMZ, TMP-sulfa, SXT, Trimethoprim/ sulfamethoxazole, Co-trimoxazole, bactrim-DS, Ciplin-DS]?

- ☐ No 0 (→ Q55)
- ☐ Yes 1
- ☐ Don't Know 997 (→ Q55)
- ☐ Refused 998 (→ Q55)

54a. When did you start taking TMP-SMX?
(Estimate okay): ___ ___ / ___ ___ ___/ ___ ___ ___ ___
Day Month Year

[Range for Day: 01-31] [Range for Month: JAN-DEC] [Range for Year: 1900, 1985-present date]
PROGRAMMER NOTE: CODE 01 JAN 1900 for Don't Know (when interviewer leaves answer blank).

- If answer is 01 JAN 1900, remove logic checks

INTERVIEWER INSTRUCTION: Leave as 01 JAN 1900 for Don't Know; if participant knows YEAR only, code 01 JAN YEAR

54b. Have you taken TMP-SMX in the past 30 days?

- ☐ No 0
- ☐ Yes 1
- ☐ Don't Know 997
- ☐ Refused 998

HIV TESTING OF SPOUSE / SEXUAL PARTNERS

INTERVIEWER READ: I would like to ask some questions about whether your spouse/sexual partners have been tested for HIV.

55. When was the last time your main MSM partner was tested for HIV?

☐ Never	0
☐ Within the last month	1
☐ More than 1 month to 6 months ago	2
☐ More than 6 months to 1 year (12 months) ago	3
☐ More than 1 year to 2 years ago	4
☐ More than 2 years ago to 4 years ago	5
☐ More than 4 years ago	6
☐ Not applicable - no main MSM partner	996
☐ Don't know	997

INTERVIEWER INSTRUCTION: Ask question and let participant respond openly. Then fit answer into the best option. If participant reports more than one Main MSM partner, ask them to pick the primary partner.

PROGRAMMER NOTE: Ask Q56 only for married participants (Q5=1 or 10 on Demographics [**DG**])

56. When was the last time your spouse was tested for HIV?

☐ Never	0
☐ Within the last month	1
☐ More than 1 month to 6 months ago	2
☐ More than 6 months to 1 year (12 months) ago	3
☐ More than 1 year to 2 years ago	4
☐ More than 2 years ago to 4 years ago	5
☐ More than 4 years ago	6
☐ Not applicable – not married	996
☐ Don't know	997

INTERVIEWER INSTRUCTION: Ask question and let participant respond openly. Then fit answer into the best option.

PROGRAMMER NOTE: Skip to END if participant is answer to Q9 = 0 (NO)

GOVERNMENT ART CENTER BOOK CONFIRMATION

57. Did the participant bring his/her ART book?

☐ No 0 [END SURVEY]
☐ Yes 1

57a. According to the ART book, when did the participant first start ART?

___ ___ / ___ ___ ___ / ___ ___ ___ ___
Day Month Year

[Range for Day: 01-31] [Range for Month: JAN-DEC] [Range for Year: 1900, 1985-present date]
PROGRAMMER NOTE: CODE 01 JAN 1900 for Don't Know (when interviewer leaves answer blank).
INTERVIEWER INSTRUCTION: Leave as 01 JAN 1900 if no start date is listed in the book. If only the YEAR is listed, code as 01 JAN YEAR

57b. According to the ART book, has the participant taken ART in the past 30 days?

☐ No 0
☐ Yes 1

57c. Are the names of the medications reported in the ART book consistent with what the participant reported?

☐ No 0
☐ Yes 1 [END SURVEY]

58. According to the medical book, which medications is the participant currently on?

58a. ___ ___ [Range: 34-49]

58b. ___ ___ [Range: 34-49]

58c. ___ ___ [Range: 34-49]

INTERVIEWER INSTRUCTION: Use the codes from **CARD HIV34-47** to answer 58a-c.

The India Men's Health Initiative

Baseline: HIV Treatment Knowledge (TK)

Participant ID ___ ___ ___ ___ ___
Date ___ ___/___ ___/___ ___
Interviewer Initials ___ ___ ___

INTERVIEWER READ: The next few questions are about HIV/AIDS and medications used to treat HIV/AIDS.

1. Do you personally know anyone who is living with HIV/AIDS?
- ☐ No 0
- ☐ Yes 1
- ☐ Don't know 997
- ☐ Refused 998

2. Before today, had you heard of antiretrovirals / antiretroviral therapy / medicines that control HIV?
- ☐ No 0 (→ Q9)
- ☐ Yes 1
- ☐ Don't know 997 (→ Q9)
- ☐ Refused 998 (→ Q9)

3. Do you personally know anyone who has taken antiretrovirals for the treatment of HIV/AIDS?
- ☐ No 0
- ☐ Yes 1
- ☐ Don't know 997
- ☐ Refused 998

INTERVIEWER READ: Please indicate whether you strongly agree, agree, disagree or strongly disagree with the following statements.

	Strongly Disagree	Disagree	Agree	Strongly Agree
4. There are medicines to cure HIV/AIDS.	1	2	3	4
5. There are medicines to treat HIV/AIDS.	1 [9]	2 [9]	3	4
6. Medicines to treat HIV/AIDS (antiretrovirals) work.	1	2	3	4
7. Medicines to treat HIV/AIDS (antiretrovirals) are easily available to those who need them in your neighborhood/locality.	1	2	3	4
8. Medicines to treat HIV/AIDS (antiretrovirals) are safe.	1	2	3	4

INTERVIEWER INSTRUCTION: Use **Card TK4.**

9. Do you know of locations where you can get HIV-related medical / clinical care and treatment?
- ☐ No 0
- ☐ Yes 1
- ☐ Don't know 997
- ☐ Refused 998

10. If effective medicines to treat HIV/AIDS were easily available in your neighborhood/locality, how would it affect your decision to get an HIV test?
    - ☐ Effective medicines are easily available in my neighborhood/locality 1
    - ☐ I would be more likely to get tested 2
    - ☐ I would be less likely to get tested 3
    - ☐ It would make no difference to me 4
    - ☐ Refused 998

11. In the past 12 months, have you heard or seen any promotions for antiretroviral therapy (ART) in your community? Either through radio, television, billboards and other advertisements?
    - ☐ No 0
    - ☐ Yes 1
    - ☐ Don't know 997
    - ☐ Refused 998

12. In the past 12 months, have you (or anyone in your family) received any information materials (e.g., pamphlets) about antiretroviral therapy (ART)?
    - ☐ No 0
    - ☐ Yes 1
    - ☐ Don't know 997
    - ☐ Refused 998

13. In the past 12 months, have there been any campaigns where HIV testing and counseling was provided in your community?
    - ☐ No 0
    - ☐ Yes 1
    - ☐ Don't know 997
    - ☐ Refused 998

14. In the past 12 months, have you heard or seen any promotions for HIV testing and counseling in your community? Either through radio, television, billboards and other advertisements?
    - ☐ No 0
    - ☐ Yes 1
    - ☐ Don't know 997
    - ☐ Refused 998

15. In the past 12 months, have you (or anyone in your family) received any information materials (e.g., pamphlets) about HIV testing and counseling?
    - ☐ No 0
    - ☐ Yes 1
    - ☐ Don't know 997
    - ☐ Refused 998

Participant ID ___ ___ ___ ___ ___
Date ___ ___/___ ___/___ ___
Interviewer Initials ___ ___ ___

Baseline Substance Use and Risk Behavior (SU)

INTERVIEWER READ: The next few questions are about your use of drugs and alcohol, and your sexual behavior.

DRUG USE

1. Have you ever injected drugs for non-medicinal purposes?
   - ☐ No 0 (→ Q12)
   - ☐ Yes 1

1a. At which age, did you first inject drugs for non-medicinal purposes?
Age: ___ ___ ___ years [Acceptable range: 10-40, 997]
INTERVIEWER INSTRUCTION: If participant does not know, code 997.

INTERVIEWER READ: Please tell me all of the drugs that you have ever injected.

[drug]	a. Have you ever injected [drug]?		b. How old were you when you first injected [drug]?	c. Have you injected [drug] in the last 6 months?		d. How often did you inject [drug] in the last 6 months?
	No	Yes	Age in years: [Acceptable Range: 10-40, 997]	No	Yes	
2. Heroin/brown sugar	0 [3]	1	___ ___ ___	0 [3]	1	☐ A few times (1)<br>☐ 1-3 times a month (2)<br>☐ About once a week (3)<br>☐ 2-5 times a week (4)<br>☐ About once a day (5)<br>☐ 2-3 times a day, almost every day (6)<br>☐ 4-9 times a day, almost every day (7)<br>☐ 10+ times a day, almost every day (8)
3. Cocaine or crack	0 [4]	1	___ ___ ___	0 [4]	1	☐ A few times (1)<br>☐ 1-3 times a month (2)<br>☐ About once a week (3)<br>☐ 2-5 times a week (4)<br>☐ About once a day (5)<br>☐ 2-3 times a day, almost every day (6)<br>☐ 4-9 times a day, almost every day (7)<br>☐ 10+ times a day, almost every day (8)
4. Stimulants (e.g. Methamphetamine, amphetamines)	0 [5]	1	___ ___ ___	0 [5]	1	☐ A few times (1)<br>☐ 1-3 times a month (2)<br>☐ About once a week (3)<br>☐ 2-5 times a week (4)<br>☐ About once a day (5)<br>☐ 2-3 times a day, almost every day (6)<br>☐ 4-9 times a day, almost every day (7)<br>☐ 10+ times a day, almost every day (8)
5. Buprenorphine (e.g. ADDNOK, tidigesic, norphine)	0 [6]	1	___ ___ ___	0 [6]	1	☐ A few times (1)<br>☐ 1-3 times a month (2)<br>☐ About once a week (3)<br>☐ 2-5 times a week (4)<br>☐ About once a day (5)<br>☐ 2-3 times a day, almost every day (6)<br>☐ 4-9 times a day, almost every day (7)<br>☐ 10+ times a day, almost every day (8)
6. Allergy medicine / antihistamines (e.g. avil, phenargan)	0 [7]	1	___ ___ ___	0 [7]	1	☐ A few times (1)<br>☐ 1-3 times a month (2)<br>☐ About once a week (3)<br>☐ 2-5 times a week (4)<br>☐ About once a day (5)<br>☐ 2-3 times a day, almost every day (6)<br>☐ 4-9 times a day, almost every day (7)<br>☐ 10+ times a day, almost every day (8)

[drug]	a. Have you ever injected **[drug]**?		b. How old were you when you first injected **[drug]**?	c. Have you injected **[drug]** in the last 6 months?		d. How often did you inject **[drug]** in the last 6 months?
	No	Yes	Age in years: [Acceptable Range: 10-40, 997]	No	Yes	
7. Painkillers (e.g. spasmoproxyvon, fortwin, morphine)	0 [8]	1	___ ___ ___	0 [8]	1	☐ A few times (1)<br>☐ 1-3 times a month (2)<br>☐ About once a week (3)<br>☐ 2-5 times a week (4)<br>☐ About once a day (5)<br>☐ 2-3 times a day, almost every day (6)<br>☐ 4-9 times a day, almost every day (7)<br>☐ 10+ times a day, almost every day (8)
8. Sedatives, Tranquilizers, Anti-Anxiety Drugs (e.g. dormin, calmpose)	0 [9]	1	___ ___ ___	0 [9]	1	☐ A few times (1)<br>☐ 1-3 times a month (2)<br>☐ About once a week (3)<br>☐ 2-5 times a week (4)<br>☐ About once a day (5)<br>☐ 2-3 times a day, almost every day (6)<br>☐ 4-9 times a day, almost every day (7)<br>☐ 10+ times a day, almost every day (8)
9. Other	0 [10]	1	___ ___ ___	0 [10]	1	☐ A few times (1)<br>☐ 1-3 times a month (2)<br>☐ About once a week (3)<br>☐ 2-5 times a week (4)<br>☐ About once a day (5)<br>☐ 2-3 times a day, almost every day (6)<br>☐ 4-9 times a day, almost every day (7)<br>☐ 10+ times a day, almost every day (8)

INTERVIEWER INSTRUCTION for Q2-Q8a: Use **Cards SU2-8.** There are more drugs on the card than on the screen so use the card and for each category, ask the participant if they have injected any of the drugs listed; If they cannot read, read the names to them.
INTERVIEWER INSTRUCTION for Q9a. If the participant names a drug that is not listed anywhere on cards SU2-8, code Yes here.
INTERVIEWER INSTRUCTION for Q2-Q9b: Code 997 for Don't Know.
INTERVIEWER INSTRUCTION for Q2-9d: Use Card SU2d.
PROGRAMMER NOTE: [X] means to skip the following a-d questions and go to the next numbered question.

- Answer to option b of question 2-9 cannot be less than answer to Q1a or greater than age of participant
- If Q1a=997 then option b of question 2-9 only needs to less than age
- If Q1=1 (Yes) then all of Q2-Q9a cannot be 0 (No)
- Include interviewer instruction on each page (e.g., Card SU2 corresponds to Q2a, Card SU3 for Q3a)

10. In the last 6 months, on how many days total did you inject ANY drug?

___ ___ ___ [Acceptable range: 0-180, 997]
INTERVIEWER INSTRUCTION: Code 997 for Don't Know
PROGRAMMER NOTE: This value should not be 0 if any value for part c from Q2-9 is coded 1.
If Q10 = 0 → SKIP to Q12.

11. In the last 6 months, on days you injected, on average how many times a day did you inject?

___ ___ ___ [Acceptable range: 1-10, 997]
INTERVIEWER INSTRUCTION: Code 997 for Don't Know

12. Have you used any drugs for non-medicinal purposes by a non-injection route in the last 6 months? By non-injection route, I mean drugs that you sniffed, snorted, smoked or ingested.

- ☐ No 0 (→ Q25)
- ☐ Yes 1

INTERVIEWER READ: Please tell me all of the drugs that you used by a non-injection route in the last 6 months. We are interested in drugs that you have sniffed, snorted, smoked or ingested for non-medical purposes.

[drug]	a. Have you used **[drug]** in the last 6 months?		b. How often did you use **[drug]** in the last 6 months?
	No	Yes	
13. Marijuana (e.g. Smoke ganga)	0 [14]	1	☐ A few times (1)<br>☐ 1-3 times a month (2)<br>☐ About once a week (3)<br>☐ 2-5 times a week (4)<br>☐ About once a day (5)<br>☐ 2-3 times a day, almost every day (6)<br>☐ 4-9 times a day, almost every day (7)<br>☐ 10+ times a day, almost every day (8)
14. Heroin (Smoke/chase opium or brown sugar)	0 [15]	1	☐ A few times (1)<br>☐ 1-3 times a month (2)<br>☐ About once a week (3)<br>☐ 2-5 times a week (4)<br>☐ About once a day (5)<br>☐ 2-3 times a day, almost every day (6)<br>☐ 4-9 times a day, almost every day (7)<br>☐ 10+ times a day, almost every day (8)
15. Stimulants (e.g. smoked or taken tablets methamphetamines, amphetamines, Yaba)	0 [16]	1	☐ A few times (1)<br>☐ 1-3 times a month (2)<br>☐ About once a week (3)<br>☐ 2-5 times a week (4)<br>☐ About once a day (5)<br>☐ 2-3 times a day, almost every day (6)<br>☐ 4-9 times a day, almost every day (7)<br>☐ 10+ times a day, almost every day (8)
16. Cocaine/crack	0 [17]	1	☐ A few times (1)<br>☐ 1-3 times a month (2)<br>☐ About once a week (3)<br>☐ 2-5 times a week (4)<br>☐ About once a day (5)<br>☐ 2-3 times a day, almost every day (6)<br>☐ 4-9 times a day, almost every day (7)<br>☐ 10+ times a day, almost every day (8)
17. Hallucinogens (e.g. LSD, MDMA, ecstasy, X)	0 [18]	1	☐ A few times (1)<br>☐ 1-3 times a month (2)<br>☐ About once a week (3)<br>☐ 2-5 times a week (4)<br>☐ About once a day (5)<br>☐ 2-3 times a day, almost every day (6)<br>☐ 4-9 times a day, almost every day (7)<br>☐ 10+ times a day, almost every day (8)

[**drug**]	a. Have you used **[drug]** in the last 6 months?		b. How often did you use **[drug]** in last 6 months?
	No	Yes	
18. Inhalants / Solvents (petrol, glue)	0 [19]	1	☐ A few times (1)<br>☐ 1-3 times a month (2)<br>☐ About once a week (3)<br>☐ 2-5 times a week (4)<br>☐ About once a day (5)<br>☐ 2-3 times a day, almost every day (6)<br>☐ 4-9 times a day, almost every day (7)<br>☐ 10+ times a day, almost every day (8)
19. Buprenorphine (e.g. ADDNOK, tidi, tidigesic, norphine, sublingual)	0 [20]	1	☐ A few times (1)<br>☐ 1-3 times a month (2)<br>☐ About once a week (3)<br>☐ 2-5 times a week (4)<br>☐ About once a day (5)<br>☐ 2-3 times a day, almost every day (6)<br>☐ 4-9 times a day, almost every day (7)<br>☐ 10+ times a day, almost every day (8)
20. Allergy medicine / antihistamines (e.g. Avil, phernergan)	0 [21]	1	☐ A few times (1)<br>☐ 1-3 times a month (2)<br>☐ About once a week (3)<br>☐ 2-5 times a week (4)<br>☐ About once a day (5)<br>☐ 2-3 times a day, almost every day (6)<br>☐ 4-9 times a day, almost every day (7)<br>☐ 10+ times a day, almost every day (8)
21. Painkillers (e.g. spasmoproxyvon, morphine, fortwin)	0 [22]	1	☐ A few times (1)<br>☐ 1-3 times a month (2)<br>☐ About once a week (3)<br>☐ 2-5 times a week (4)<br>☐ About once a day (5)<br>☐ 2-3 times a day, almost every day (6)<br>☐ 4-9 times a day, almost every day (7)<br>☐ 10+ times a day, almost every day (8)
22. Sedatives, Tranquilizers, Anti-Anxiety Drugs (e.g. Dormin, Calmpose)	0 [23]	1	☐ A few times (1)<br>☐ 1-3 times a month (2)<br>☐ About once a week (3)<br>☐ 2-5 times a week (4)<br>☐ About once a day (5)<br>☐ 2-3 times a day, almost every day (6)<br>☐ 4-9 times a day, almost every day (7)<br>☐ 10+ times a day, almost every day (8)
23. Chew intoxicating tobacco (e.g. mawa, zarda)	0 [24]	1	☐ A few times (1)<br>☐ 1-3 times a month (2)<br>☐ About once a week (3)<br>☐ 2-5 times a week (4)<br>☐ About once a day (5)<br>☐ 2-3 times a day, almost every day (6)<br>☐ 4-9 times a day, almost every day (7)<br>☐ 10+ times a day, almost every day (8)

	a. Have you used **[drug]** in the last 6 months?		b. How often did you use **[drug]** in the last 6 months?
	No	Yes	
24. Any other drugs	0 [25]	1	☐ A few times (1)<br>☐ 1-3 times a month (2)<br>☐ About once a week (3)<br>☐ 2-5 times a week (4)<br>☐ About once a day (5)<br>☐ 2-3 times a day, almost every day (6)<br>☐ 4-9 times a day, almost every day (7)<br>☐ 10+ times a day, almost every day (8)

INTERVIEWER INSTRUCTION for **Q13-Q24a**: Use **Card SU13-24.**
- There are more drugs on the card than on the screen so use the card and for each category, ask the participant if they have injected any of the drugs listed; If they cannot read, read the names to them.
- **INTERVIEWER INSTRUCTION for Q24a.** Use Card SU24. If participant names a drug on Card SU24 or a drug that is not listed anywhere on cards SU13-24, code Yes here.
- **INTERVIEWER INSTRUCTION** for **Q13-24b:** Use Card SU2d.

PROGRAMMER NOTE: [X] means to skip part b of the question, and go onto the next numbered question.
- If Q12 = 1 (Yes) then at least one of Q13a-Q24a has to be 1
- Include interviewer instruction on each page (e.g., Card SU13 corresponds to Q13a; Card SU14 corresponds to Q14a)

AUDIT: ALCOHOL USE

25. How often do you have a drink containing alcohol?
- ☐ Never 0 (→ Q35)
- ☐ 1 time a month, or less 1
- ☐ 2–4 times a month 2
- ☐ 2–3 times a week 3
- ☐ 4 or more times a week 4

INTERVIEWER INSTRUCTION: Use **Card SU25**

26. How many standard drinks containing alcohol do you have on a typical day when drinking?
- ☐ 1 or 2 0
- ☐ 3 or 4 1
- ☐ 5 or 6 2
- ☐ 7 to 9 3
- ☐ 10 or more 4

INTERVIEWER INSTRUCTION: Use **Card SU26** to translate number of drinks.

27. How often do you have six or more drinks on one occasion?
- ☐ Never 0
- ☐ Less than monthly 1
- ☐ Monthly 2
- ☐ Weekly 3
- ☐ Daily / Almost daily 4

INTERVIEWER INSTRUCTION: Use **Card SU27**

28. During the past year, how often have you found that you were not able to stop drinking once you had started?
    - ☐ Never 0
    - ☐ Less than monthly 1
    - ☐ Monthly 2
    - ☐ Weekly 3
    - ☐ Daily / Almost daily 4

INTERVIEWER INSTRUCTION: Use **Card SU27**

29. During the past year, how often have you failed to do what was normally expected of you because of drinking?
    - ☐ Never 0
    - ☐ Less than monthly 1
    - ☐ Monthly 2
    - ☐ Weekly 3
    - ☐ Daily / Almost daily 4

INTERVIEWER INSTRUCTION: Use **Card SU27**

30. During the past year, how often have you needed a drink in the morning to get yourself going after a heavy drinking session?
    - ☐ Never 0
    - ☐ Less than monthly 1
    - ☐ Monthly 2
    - ☐ Weekly 3
    - ☐ Daily / Almost daily 4

INTERVIEWER INSTRUCTION: Use **Card SU27**

31. During the past year, how often have you had a feeling of guilt or remorse after drinking?
    - ☐ Never 0
    - ☐ Less than monthly 1
    - ☐ Monthly 2
    - ☐ Weekly 3
    - ☐ Daily / Almost daily 4

INTERVIEWER INSTRUCTION: Use **Card SU27**

32. During the past year, have you been unable to remember what happened the night before because you had been drinking?
    - ☐ Never 0
    - ☐ Less than monthly 1
    - ☐ Monthly 2
    - ☐ Weekly 3
    - ☐ Daily / Almost daily 4

INTERVIEWER INSTRUCTION: Use **Card SU27**

33. Have you or someone else been injured as a result of your drinking?
    - ☐ No 0
    - ☐ Yes, but not in the past year 2
    - ☐ Yes, during the past year 4

INTERVIEWER INSTRUCTION: Use **Card SU33**

34. Has a relative or friend, doctor or other health worker been concerned about your drinking or suggested you cut down?
    - ☐ No 0
    - ☐ Yes, but not in the past year 2
    - ☐ Yes, during the past year 4

INTERVIEWER INSTRUCTION: Use **Card SU33**

RISK BEHAVIOR

PROGRAMMER NOTE: Skip Questions 35-39 if Q1 = 0

35. Have you ever passed a needle or syringe to someone else after you used it?
    - ☐ No 0 (→ Q36)
    - ☐ Yes 1

    35a. When was the last time you did this?
    - ☐ Within the past 30 days 1
    - ☐ More than 1 month to 3 months ago 2 (→ Q36)
    - ☐ More than 3 months to 6 months ago 3 (→ Q36)
    - ☐ More that 6 months to 1 year (12 months) ago 4 (→ Q36)
    - ☐ More than 1 year ago 5 (→ Q36)

    35b. In the last 30 days, to how many PERSONS did you pass a needle or syringe to after you used it?

    ___ ___ ___ [Acceptable range: 1-30, 997]

    INTERVIEWER INSTRUCTION: Code 997 for Don't Know.

    35c. In the last 30 days, how many TIMES did you pass a needle or syringe to someone after you used it?

    ___ ___ ___ [Acceptable range: 1-180, 997]

    INTERVIEWER INSTRUCTION: Code 997 for Don't Know.

36. Have you ever used a needle or syringe after someone else used it?
    - ☐ No 0 (→ Q37)
    - ☐ Yes 1

    36a. When was the last time you did this?
    - ☐ Within the past 30 days 1
    - ☐ More than 1 month to 3 months ago 2 (→ Q37)
    - ☐ More than 3 months to 6 months ago 3 (→ Q37)
    - ☐ More that 6 months to 1 year (12 months) ago 4 (→ Q37)
    - ☐ More than 1 year ago 5 (→ Q37)

    36b. In the last 30 days, how many PERSONS used a needle or syringe before you used it?

    ___ ___ ___ [Acceptable range: 1-30, 997]

    INTERVIEWER INSTRUCTION: Code 997 for Don't Know.

    36c. In the last 30 days, how many of these PERSONS were known to you to be HIV positive?

    ___ ___ ___ [Acceptable range: 0-30, 997]

    INTERVIEWER INSTRUCTION: Code 997 for Don't Know.

    PROGRAMMER NOTE: Q36c cannot be greater than Q36b.

36d. In the last 30 days, how many TIMES did you use a needle or syringe after someone else used it?
___ ___ ___ [Acceptable range: 1-180, 997]
INTERVIEWER INSTRUCTION: Code 997 for Don't Know.

36e. In the last 30 days, how many TIMES did you use a needle or syringe after an HIV positive person used it?

___ ___ ___ [Acceptable range: 0-180, 997]
INTERVIEWER INSTRUCTION: Code 997 for Don't Know.
PROGRAMMER NOTE: Q36e cannot be greater than Q36d.

37. Think of the last time you injected drugs. Did you share a needle or syringe?
- ☐ No 0
- ☐ Yes 1

PROGRAMMER NOTE: Ask Q38 and Q39 ONLY if participant injected at least once in last 6 months: At least one of Q2c-Q9c =1. If all Q2c-Q9c = 0 or missing, skip to Q40.
38. In the last 6 months, think about who you injected with. How often did you inject [**a-d**]?

	**Never**	**Less than half the time**	**Half of the time**	**More than half the time**	**Always**	**Refused**
38a. alone	0	1	2	3	4	998
38b. with spouse/ sexual partner	0	1	2	3	4	998
38c. with one person (other than a spouse/ partner)	0	1	2	3	4	998
38d. with multiple other persons	0	1	2	3	4	998

INTERVIEWER INSTRUCTION: Use Card SU38.
PROGRAMMER NOTE: Participant can only pick Always (4) or More than Half the Time (3) once for 38a-d.
- Include, "How often did you inject." on each page.

39. In the last six months, in which places did you inject drugs? (**SELECT ALL THAT APPLY**)

	**Yes**	**No**
39a. Home	1	0
39b. Friend's house	1	0
39c. Dealer's place	1	0
39d. Public park/playground	1	0
39e. Public toilet	1	0
39f. Shooting gallery	1	0
39g. Graveyard, cemetery, burial ground	1	0
39h. Other	1	0
39i. Don't Know	1	0
39j. Refused	1	0

INTERVIEWER INSTRUCTION: Please read all choices a-h to participant.
PROGRAMMER NOTE: Q39a-39j should be on one page.
- If option '39i or 39j' is selected then all of options '39a-39h' should be set to missing.

SEXUAL BEHAVIOR

INTERVIEWER READ: In the next set of questions, we are going to ask you about your sexual behavior. These questions may be difficult to answer. Please remember that your name will not be connected to any of this information. There are no right or wrong answers. Please be honest when you answer. It is critical to our understanding about HIV and sexual behavior that you answer truthfully. For all questions, if you do not know the exact number for an answer, please make your best guess.

40. At what age did you first have oral sex with a man or hijra?

___ ___ ___ [Acceptable Range: 8-60, 997]
INTERVIEWER INSTRUCTION: Code 997 for Don't Know
PROGRAMMER NOTE: Q40 cannot be greater than age from Q1 of Demographics

40a. At what age did you first have insertive anal sex with a man or hijra? (Insertive anal sex means you put your penis in the anus of another man/hijra)

___ ___ ___ [Acceptable Range: 8-60, 995, 997]
INTERVIEWER INSTRUCTION: Code 995 for Never had insertive anal sex.
Code 997 for Don't Know.
PROGRAMMER NOTE: Q40a cannot be greater than age from Q1 of Demographics.

40b. At what age did you first have receptive anal sex with a man? (Receptive anal sex means some other man has inserted his penis in your anus)

___ ___ ___ [Acceptable Range: 8-60, 995, 997]
INTERVIEWER INSTRUCTION: Code 995 for Never had receptive anal sex.
Code 997 for Don't Know.
PROGRAMMER NOTE: Q40b cannot be greater than age from Q1 of Demographics.

40c. How many male sexual partners have you had oral or anal sex with in your lifetime?

___ ___ ___ [Range: 1-995, 997]
INTERVIEWER INSTRUCTION: Code 997 for Don't Know.
Code 995 if more than 995.

41. In the last 6 months, with how many different men or hijra have you had oral sex?

___ ___ ___ [Acceptable Range: 0-180]
PROGRAMMER NOTE: Answer to Q41 must be less than Q40c

42. In the last 6 months, with how many different men or hijra have you had anal sex?

___ ___ ___ [Acceptable Range: 0-180]
PROGRAMMER NOTE: If 0, SKIP TO Q49.
PROGRAMMER NOTE: Answer to Q42 must be less than Q40C

INTERVIEWER READ: In the next set of questions, we will talk about your most recent male sexual partner(s) and I will ask you to tell me the following things about them: their relationship with you (boyfriend, friend, sex worker, etc.), how often you have had anal sex with this partner during the past 6 months, and whether or not you used condoms with this partner. I am interested only in the last 4 partners that you had anal sex with.

READ IF NUMBER OF PARTNERS IS 1-4:
You mentioned that you had [**number of partners from Q42**] male sexual partners with whom you had anal sex with in the last 6 months. Let's start with the most recent partner.

READ IF NUMBER OF PARTNERS IS >4:
You mentioned that you had [**number of partners from Q42**] male sexual partners with whom you had anal sex with in the last 6 months. Even though you have told me that you have had anal sex with more than four male partners in the last 6 months, let's focus on the 4 most recent partners. Let's start with the most recent partner.

PROGRAMMER NOTE: Prepopulate number of partners with Answer to Q42.

- Only show instruction 'READ IF NUMBER OF PARTNERS IS 1-4' if Q42≤4.
- Only show instruction 'READ IF NUMBER OF PARTNERS IS >4' if Q42>4.

Most recent partner first:		***43. Partner #1***	***44. Partner #2***	***45. Partner #3***	***46. Partner #4***
a.	What are the initials or nickname of your male sexual partner?	______	______	______	______
b.	Is [**this male partner**] kothi, panthi, DD, gay, bisexual or hijra?	☐ Kothi (1)<br>☐ Panthi (2)<br>☐ DD(3)<br>☐ Gay (4)<br>☐ Bisexual (5)<br>☐ Hijra(6)<br>☐ MSM (7)<br>☐ Other (996)	☐ Kothi (1)<br>☐ Panthi (2)<br>☐ DD(3)<br>☐ Gay (4)<br>☐ Bisexual (5)<br>☐ Hijra(6)<br>☐ MSM (7)<br>☐ Other (996)	☐ Kothi (1)<br>☐ Panthi (2)<br>☐ DD(3)<br>☐ Gay (4)<br>☐ Bisexual (5)<br>☐ Hijra(6)<br>☐ MSM (7)<br>☐ Other (996)	☐ Kothi (1)<br>☐ Panthi (2)<br>☐ DD(3)<br>☐ Gay (4)<br>☐ Bisexual (5)<br>☐ Hijra(6)<br>☐ MSM (7)<br>☐ Other (996)
c	What relationship do you have with [**this male partner**]?	☐ Boyfriend (2)<br>☐ Casual partner / friend (3)<br>☐ Commercial sex worker (4)<br>☐ One time partner (5)<br>☐ Other (996)	☐ Boyfriend (2)<br>☐ Casual partner / friend (3)<br>☐ Commercial sex worker (4)<br>☐ One time partner (5)<br>☐ Other (996)	☐ Boyfriend (2)<br>☐ Casual partner / friend (3)<br>☐ Commercial sex worker (4)<br>☐ One time partner (5)<br>☐ Other (996)	☐ Boyfriend (2)<br>☐ Casual partner / friend (3)<br>☐ Commercial sex worker (4)<br>☐ One time partner (5)<br>☐ Other (996)
d.	Was [**this male partner**] HIV-positive?	☐ No (0)<br>☐ Yes (1)<br>☐ DK (997)	☐ No (0)<br>☐ Yes (1)<br>☐ DK (997)	☐ No (0)<br>☐ Yes (1)<br>☐ DK (997)	☐ No (0)<br>☐ Yes (1)<br>☐ DK (997)
e.	How often have you used alcohol or drugs before you had sex with [**this male partner**] in the last six months?	☐ Never (0)<br>☐ Sometimes (1)<br>☐ Always (2)	☐ Never (0)<br>☐ Sometimes (1)<br>☐ Always (2)	☐ Never (0)<br>☐ Sometimes (1)<br>☐ Always (2)	☐ Never (0)<br>☐ Sometimes (1)<br>☐ Always (2)
f.	How often have you had insertive anal sex with [**this male partner**] in the last six months?<br><br>**INTERVIEWER INSTRUCTION:** Use Card SU43.	☐ Never (0)<br>☐ Once or twice a month or less often (1)<br>☐ 2-3 times a month (2)<br>☐ Once a week<br>☐ 2-4 times per week (3)<br>☐ More than 4 times per week (4)<br>If 0, skip to Q43i.	☐ Never (0)<br>☐ Once or twice a month or less often (1)<br>☐ 2-3 times a month (2)<br>☐ Once a week<br>☐ 2-4 times per week (3)<br>☐ More than 4 times per week (4)<br>If 0, skip to Q44i.	☐ Never (0)<br>☐ Once or twice a month or less often (1)<br>☐ 2-3 times a month (2)<br>☐ Once a week<br>☐ 2-4 times per week (3)<br>☐ More than 4 times per week (4)<br>If 0, skip to Q45i.	☐ Never (0)<br>☐ Once or twice a month or less often (1)<br>☐ 2-3 times a month (2)<br>☐ Once a week<br>☐ 2-4 times per week (3)<br>☐ More than 4 times per week (4)<br>If 0, skip to Q46i.
g.	How often have you used a condom during insertive anal sex with [**this male partner**] in the last six months?	☐ Never (0)<br>☐ Sometimes (1)<br>☐ Always (2)<br>If 0 skip to 43i.	☐ Never (0)<br>☐ Sometimes (1)<br>☐ Always (2)<br>If 0 skip to 44i.	☐ Never (0)<br>☐ Sometimes (1)<br>☐ Always (2)<br>If 0 skip to 45i.	☐ Never (0)<br>☐ Sometimes (1)<br>☐ Always (2)<br>If 0 skip to 46i.
h.	Did you use a condom the last time you had insertive anal sex with [**this male partner**]?	☐ No (0)<br>☐ Yes (1)	☐ No (0)<br>☐ Yes (1)	☐ No (0)<br>☐ Yes (1)<br>.	☐ No (0)<br>☐ Yes (1)

i.	How often have you had receptive anal sex with [**this male partner**] in the last six months?<br><br>**INTERVIEWER INSTRUCTION:** Use Card SU43.	☐ Never (0)<br>☐ Once or twice a month or less often (1)<br>☐ 2-3 times a month (2)<br>☐ 2-4 times per week (3)<br>☐ More than 4 times per week (4)<br>☐ DK (997)<br>If 0 & Q42>1, skip to Q44a.<br>If 0 & Q42=1, skip to Q49.	☐ Never (0)<br>☐ Once or twice a month or less often (1)<br>☐ 2-3 times a month (2)<br>☐ 2-4 times per week (3)<br>☐ More than 4 times per week (4)<br>☐ DK (997)<br>If 0 & Q42>1, skip to Q45a.<br>If 0 & Q42=2, skip to Q49.	☐ Never (0)<br>☐ Once or twice a month or less often (1)<br>☐ 2-3 times a month (2)<br>☐ 2-4 times per week (3)<br>☐ More than 4 times pe.r week (4)<br>☐ DK (997)<br>If 0 & Q42>1, skip to Q46a.<br>If 0 & Q42=3, skip to Q49.	☐ Never (0)<br>☐ Once or twice a month or less often (1)<br>☐ 2-3 times a month (2)<br>☐ 2-4 times per week (3)<br>☐ More than 4 times per week (4)<br>☐ DK (997)<br>If 0 & Q42>1, skip to Q47.<br>If 0 & Q42=4, skip to Q49.
j.	How often have you used a condom during receptive anal sex with [**this male partner**] in the last six months?	☐ Never (0)<br>☐ Sometimes (1)<br>☐ Always (2)<br>If 0 & Q42>1, skip to Q44a.<br>If 0 & Q42=1, skip to Q49.	☐ Never (0)<br>☐ Sometimes (1)<br>☐ Always (2)<br>If 0 & Q42>2, skip to Q45a.<br>If 0 & Q42=2, skip to Q49.	☐ Never (0)<br>☐ Sometimes (1)<br>☐ Always (2)<br>If 0 & Q42>3, skip to Q46a.<br>If 0 & Q42=3, skip to Q49.	☐ Never (0)<br>☐ Sometimes (1)<br>☐ Always (2)<br>If 0 & Q42>4, skip to Q47.<br>If 0 & Q42=4, skip to Q49.
k.	Did you use a condom the last time you had receptive anal sex with [**this male partner**]?	☐ No (0)<br>☐ Yes (1)<br>If Q42=1, skip to Q49.	☐ No (0)<br>☐ Yes (1)<br>If Q42=2, skip to Q49.	☐ No (0)<br>☐ Yes (1)<br>If Q42=3, skip to Q49.	☐ No (0)<br>☐ Yes (1)<br>If Q42=4, skip to Q49.

PROGRAMMER NOTE: Pre-populate [this male partner] in parts 'b-k' with initials/nickname from part 'a' for each question Q43-46.

- For Q43-46, if f=0 and i=0, add a pop-up box after I that says 'Both f and I cannot be 'Never' as you said you had sex with this partner in the past 6 months.'
- For Q43-46, if b = 6 (hijra) then skip i-k
- For Q43-36, If c = 5 (one time partner) then f and i must be 0 or 1

PROGRAMMER NOTE: Only ask Questions 47-48 for those whose answer to Q42>4. If Q42≤4, SKIP TO Q49.

INTERVIEWER READ: Earlier you said you've had anal sex with **[# partners from Q42]** male partners in the past 6 months. I want to ask more details about all of these partners.

PROGRAMMER NOTE: Prepopulate number of partners with Answer to Q42

47. How many of these men were "regular partners"? By "regular partner", I mean a man who you have sex with and who you feel committed to above most people.

___ ___ ___ [Acceptable Range: 0-180,997]
PROGRAMMER NOTE: Number must be less than or equal to Q42.
If 0, skip to Q48.
INTERVIEWER INSTRUCTION: Code 997 for Don't Know

47a. How often did you use a condom with these regular male partners?

☐ Never 1
☐ Sometimes 2
☐ Always 3

48. How many of these men were "casual or exchange partners"? By "casual partner", I mean a man who you have sex with but do not feel committed to or don't know very well. And by exchange partners, I mean a man you have sex with in exchange for things like money or drugs.

___ ___ ___ [Acceptable Range: 0-180, 997]
PROGRAMMER NOTE: Number must be less than or equal to Q42.
PROGRAMMER NOTE: Prepopulate the field with Q42-Q47 and the interviewer can just check with the participant to make sure this is correct
If 0, skip to Q49.
INTERVIEWER INSTRUCTION: Code 997 for Don't Know
- This field will be pre-filled according to the responses the participant gave to previous questions.

48a. How often did you use a condom with these casual male partners?
- ☐ Never 1
- ☐ Sometimes 2
- ☐ Always 3

INTERVIEWER READ: Now I would like to ask you about your female partners including your wife.

49. Have you ever had vaginal or anal sex with a woman (including wife)?
- ☐ No 0 (→ Q58)
- ☐ Yes 1

50. At what age did you first have vaginal or anal sex with a woman?

___ ___ ___ [Acceptable Range: 8-60, 997]
INTERVIEWER INSTRUCTION: Code 997 for Don't Know

50a. In your lifetime, with how many women have you had vaginal or anal sex?
___ ___ ___ [Range: 1-995, 997]
INTERVIEWER INSTRUCTION: Code 995 for more than 995 partners; Code 997 for Don't Know

51. In the last 6 months, with how many different women have you had vaginal or anal sex?

___ ___ ___ [Acceptable Range: 0-180]
PROGRAMMER NOTE: IF Q51=0, skip to Q58

INTERVIEWER READ: In the next set of questions, we will talk about your most recent female sexual partner(s) and I will ask you to tell me the following things about them: their relationship with you (spouse, friend, sex worker, etc.), how many times you've had vaginal or anal sex with this partner during the past 6 months, and whether or not you used condoms with this partner.

IF NUMBER OF PARTNERS IS 1-4: You mentioned that had [**number of partners from Q51**] female sexual partners in the past 6 months. Let's start with the most recent partner.

IF NUMBER OF PARTNERS IS >4: You mentioned that had [**number of partners from Q51**] female sexual partners in the past 6 months. Even though you have told me that you have had more than four female partners in the past 6 months, let's focus on the 4 most recent partners. Let's start with the most recent partner.

PROGRAMMER NOTE: Prepopulate number of partners with the number from Q51.
- Only show instruction 'READ IF NUMBER OF PARTNERS IS 1-4 if Q51≤4'
- Only show instruction 'READ IF NUMBER OF PARTNERS IS >4 if Q51>4'

Most recent partner first:		***52. Partner #1***	***53. Partner #2***	***54. Partner #3***	***55. Partner #4***
a.	What are the initials or nickname of your female sexual partner?	________	________	________	________
b.	What relationship do you have with [**this female partner**]?	☐ Spouse (1)<br>☐ Girlfriend (2)<br>☐ Casual partner / friend (3)<br>☐ Commercial sex worker (4)<br>☐ One time partner (5)<br>☐ Other (996)	☐ Spouse (1)<br>☐ Girlfriend (2)<br>☐ Casual partner / friend (3)<br>☐ Commercial sex worker (4)<br>☐ One time partner (5)<br>☐ Other (996)	☐ Spouse (1)<br>☐ Girlfriend (2)<br>☐ Casual partner / friend (3)<br>☐ Commercial sex worker (4)<br>☐ One time partner (5)<br>☐ Other (996)	☐ Spouse (1)<br>☐ Girlfriend (2)<br>☐ Casual partner / friend (3)<br>☐ Commercial sex worker (4)<br>☐ One time partner (5)<br>☐ Other (996)
c.	Was [**this female partner**] HIV-positive?	☐ No (0)<br>☐ Yes (1)<br>☐ DK (997)	☐ No (0)<br>☐ Yes (1)<br>☐ DK (997)	☐ No (0)<br>☐ Yes (1)<br>☐ DK (997)	☐ No (0)<br>☐ Yes (1)<br>☐ DK (997)
d.	How often have you had vaginal sex with [**this female partner**] in the past six months?<br><br>**INTERVIEWER INSTRUCTION:** Use Card SU43.	☐ Never (0)<br>☐ Once or twice a month or less often (1)<br>☐ 2-3 times a month (2)<br>☐ 2-4 times per week (3)<br>☐ More than 4 times per week (4)<br>☐ DK (997)<br>If 0, skip to Q52g	☐ Never (0)<br>☐ Once or twice a month or less often (1)<br>☐ 2-3 times a month (2)<br>☐ 2-4 times per week (3)<br>☐ More than 4 times per week (4)<br>☐ DK (997)<br>If 0, skip to Q53g	☐ Never (0)<br>☐ Once or twice a month or less often (1)<br>☐ 2-3 times a month (2)<br>☐ 2-4 times per week (3)<br>☐ More than 4 times per week (4)<br>☐ DK (997)<br>If 0, skip to Q54g	☐ Never (0)<br>☐ Once or twice a month or less often (1)<br>☐ 2-3 times a month (2)<br>☐ 2-4 times per week (3)<br>☐ More than 4 times per week (4)<br>☐ DK (997)<br>If 0, skip to Q55g
e.	How often have you used a condom during vaginal sex with [**this female partner**] in the past six months?	☐ Never (0)<br>☐ Sometimes (1)<br>☐ Always (2)<br>If 0, skip to Q52g	☐ Never (0)<br>☐ Sometimes (1)<br>☐ Always (2)<br>If 0, skip to Q53g	☐ Never (0)<br>☐ Sometimes (1)<br>☐ Always (2)<br>If 0, skip to Q54g	☐ Never (0)<br>☐ Sometimes (1)<br>☐ Always (2)<br>If 0, skip to Q55g
f.	Did you use a condom the last time you had vaginal sex with [**this female partner**]?	☐ No (0)<br>☐ Yes (1)	☐ No (0)<br>☐ Yes (1)	☐ No (0)<br>☐ Yes (1)	☐ No (0)<br>☐ Yes (1)
g.	How often have you had anal sex with [**this female partner**] in the past six months?<br><br>**INTERVIEWER INSTRUCTION:** Use Card SU43.	☐ Never (0)<br>☐ Once or twice a month or less often (1)<br>☐ 2-3 times a month (2)<br>☐ 2-4 times per week (3)<br>☐ More than 4 times per week (4)<br>☐ DK (997)<br>If 0 & Q51>1, skip to Q53a.<br>If 0 & Q51=1, skip to Q58.	☐ Never (0)<br>☐ Once or twice a month or less often (1)<br>☐ 2-3 times a month (2)<br>☐ 2-4 times per week (3)<br>☐ More than 4 times per week (4)<br>☐ DK (997)<br>If 0 & Q51>2, skip to Q54a.<br>If 0 & Q51=2, skip to Q58.	☐ Never (0)<br>☐ Once or twice a month or less often (1)<br>☐ 2-3 times a month (2)<br>☐ 2-4 times per week (3)<br>☐ More than 4 times per week (4)<br>☐ DK (997)<br>If 0 & Q51>3, skip to Q55a.<br>If 0 & Q51=3, skip to Q58.	☐ Never (0)<br>☐ Once or twice a month or less often (1)<br>☐ 2-3 times a month (2)<br>☐ 2-4 times per week (3)<br>☐ More than 4 times per week (4)<br>☐ DK (997)<br>If 0 & Q51>4, skip to Q56.<br>If 0 & Q51=4, skip to Q58.

h.	How often have you used a condom during anal sex with [**this female partner**] in the past six months?	☐ Never (0)<br>☐ Sometimes (1)<br>☐ Always (2)<br>If 0 & Q51>1, skip to Q53a.<br>If 0 & Q51=1, skip to Q58.	☐ Never (0)<br>☐ Sometimes (1)<br>☐ Always (2)<br>If 0 & Q51>2, skip to Q54a.<br>If 0 & Q51=2, skip to Q58.	☐ Never (0)<br>☐ Sometimes (1)<br>☐ Always (2)<br>If 0 & Q51>3, skip to Q55a.<br>If 0 & Q51=3, skip to Q58.	☐ Never (0)<br>☐ Sometimes (1)<br>☐ Always (2)<br>If 0 & Q51>4, skip to Q56.<br>If 0 & Q51=4, skip to Q58.
i.	Did you use a condom the last time you had anal sex with [**this female partner**]?	☐ No (0)<br>☐ Yes (1)<br>If Q51=1, skip to Q58.	☐ No (0)<br>☐ Yes (1)<br>If Q51=2, skip to Q58.	☐ No (0)<br>☐ Yes (1)<br>If Q51=3, skip to Q58.	☐ No (0)<br>☐ Yes (1)<br>If Q51=4, skip to Q58.

PROGRAMMER NOTE: Pre-populate [this female partner] in parts 'b-I' with initials/nickname from part 'a' for each question Q52-55.

- For Q52-55, if d=0 and g=0, add a pop-up box after g that says 'Both d and g cannot be 'Never' as you said you had sex with this partner in the past 6 months.'
- For Q52-55, If b = 5 (one time partner) then d and g must be 0 or 1

PROGRAMMER NOTE: Only ask Questions 56-57 for those whose answer to Q51>4. If Q51≤4, SKIP TO Q58.

INTERVIEWER READ: Earlier you said you've had sex with **[# partners from Q51]** female partners in the past 6 months. I want to ask more details about all of these partners.

PROGRAMMER NOTE: Prepopulate number of partners with answer to Q51

56. How many of these women were "regular partners"? By "regular partner", I mean a woman who you have sex with and who you feel committed to above most people.

___ ___ ___ [Acceptable Range: 0-180,997]
PROGRAMMER NOTE: Number must be less than or equal to Q51.
If 0, skip to Q57.

INTERVIEWER INSTRUCTION: Code 997 for Don't Know

56a. How often did you use a condom with these regular female partners?

☐ Never 1
☐ Sometimes 2
☐ Always 3

57. How many of these women were "casual or exchange partners"? By "casual partner", I mean a woman who you have sex with but do not feel committed to or don't know very well. And by exchange partners, I mean a woman you have sex with in exchange for things like money or drugs.

___ ___ ___ [Acceptable Range: 0-180, 997]
PROGRAMMER NOTE: Number must be less than or equal to Q51
PROGRAMMER NOTE: Prepopulate the field with Q51-Q56 and the interviewer can just check with the participant to make sure this is correct
If 0, skip to Q58.

INTERVIEWER INSTRUCTION: Code 997 for Don't Know

- This field will be pre-filled according to the responses the participant gave to previous questions.

57a. How often did you use a condom with these casual female partners?
- ☐ Never 1
- ☐ Sometimes 2
- ☐ Always 3

58. Have you ever had sex to receive money, alcohol, drugs or other things?
- ☐ No 0 (→Q59)
- ☐ Yes 1
- ☐ Don't know 997 (→Q59)

58a. When was the last time you did this?
- ☐ Within the past 30 days 1
- ☐ More than 1 month to 3 months ago 2
- ☐ More than 3 months to 6 months ago 3
- ☐ More that 6 months to 1 year (12 months) ago 4
- ☐ More than 1 year ago 5

59. Have you ever given money, alcohol, or drugs for sex?
- ☐ No 0 (→END)
- ☐ Yes 1
- ☐ Don't know 997 (→END)

59a. When was the last time you did this?
- ☐ Within the past 30 days 1
- ☐ More than 1 month to 3 months ago 2
- ☐ More than 3 months to 6 months ago 3
- ☐ More that 6 months to 1 year (12 months) ago 4
- ☐ More than 1 year ago 5

The India Men's Health Initiative

Baseline Services (SV)

Participant ID ___ ___ ___ ___ ___
Date ___ ___/___ ___/___ ___
Interviewer Initials ___ ___ ___

INTERVIEWER READ: The next questions are about your experiences with services

PROGRAMMER NOTE: Skip to Q13 if the participant has never injected drugs (Q1 on **SU** = 0).

1. When did you last visit a needle exchange program?
   - ☐ Never 0
   - ☐ Within the last month 1 (→ Q4)
   - ☐ More than 1 month to 6 months ago 2 (→ Q4)
   - ☐ More than 6 months to 1 year (12 months) ago 3 (→ Q3)
   - ☐ More than 1 year to 2 years ago 4 (→ Q3)
   - ☐ More than 2 years ago to 4 years ago 5 (→ Q3)
   - ☐ More than 4 years ago 6 (→ Q3)
   - ☐ Don't know 997 (→ Q6)

INTERVIEWER INSTRUCTION: Ask question and let participant respond openly. Then fit answer into best option.

2. Why have you never gotten needles from a needle exchange program? (**SELECT ALL THAT APPLY**)

	**Yes**	**No**
2a. I inject very infrequently	1	0
2b. I do not know where to find a needle exchange program	1	0
2c. The programs are too far away/ difficult to get to	1	0
2d. I do not have time to go	1	0
2e. The hours of operation of the programs are inconvenient for me	1	0
2f. None of my friends/network partners go	1	0
2g. My friends always bring needles	1	0
2h. I get needles from the pharmacy	1	0
2i. Friends/network partners have been treated badly at needle exchange programs	1	0
2j. I am worried I will lose my job or house if people find out I am an injection drug user	1	0
2k. I am afraid of incarceration / the authorities	1	0
2l. Don't Know	1 [6]	0
2m. Refused	1 [6]	0

INTERVIEWER INSTRUCTION: Please read All Choices a-m to Participant; Use **Card SV2**.

PROGRAMMER NOTE: Q2a-2m should be on one page

- If any of 'a-k' is selected at the same time as 'l' or 'm' then a pop-up box should appear 'You should not select DON'T KNOW/REFUSED when you have selected any other options. Please check answers.'

2a1. Which of these is the primary reason you have NEVER gotten needles from a needle exchange program?
   - ☐ I inject very infrequently 1
   - ☐ I do not know where to find a needle exchange program 2
   - ☐ The programs are too far away / difficult to access 3
   - ☐ I do not have time to go 4
   - ☐ The hours of operation of the programs are inconvenient for me 5
   - ☐ None of my friends/network partners go 6
   - ☐ My friends always bring needles 7
   - ☐ I get needles from the pharmacy 8
   - ☐ Friends/network partners have been treated badly at needle exchange programs 9
   - ☐ I am worried I will lose my job or house if people find out I am an injection drug user 10
   - ☐ I am afraid of incarceration / the authorities 11

PROGRAMMER NOTE: Skip to Q6
- Only a response from Q2 that is coded as 1 'YES' can be selected here.

3. Why have you not gotten needles from a needle exchange program in the last six months? (**SELECT ALL THAT APPLY**)

	Yes	No
3a. I have not injected in the past six months	1	0
3b. I do not know where to find a needle exchange program	1	0
3c. The programs are too far away/ difficult to get to	1	0
3d. I do not have time to go	1	0
3e. The hours of operation of the programs are inconvenient for me	1	0
3f. None of my friends/network partners go	1	0
3g. My friends always bring needles	1	0
3h. I get needles from the pharmacy	1	0
3i. I have been treated badly at needle exchange programs	1	0
3j. Friends/network partners have been treated badly at needle exchange programs	1	0
3k. I am worried I will lose my job or house if people find out I am an injection drug user	1	0
3l. Don't Know	1 [4]	0
3m. Refused	1 [4]	0

INTERVIEWER INSTRUCTION: Please read All Choices a-k to Participant; Use **Card SV3**.
PROGRAMMER NOTE: Q3a-3m should be on one page
- If any of 'a-k' is selected at the same time as 'l' or 'm' then a pop-up box should appear 'You should not select DON'T KNOW/REFUSED when you have selected any other options. Please check answers.'

3a1. Which of these is the primary reason you haven't gotten needles from a needle exchange program in the last six months?

- ☐ I have not injected in the past six months 1
- ☐ I do not know where to find a needle exchange program 2
- ☐ The programs are too far away / difficult to access 3
- ☐ I do not have time to go 4
- ☐ The hours of operation of the programs are inconvenient for me 5
- ☐ None of my friends/network partners go 6
- ☐ My friends always bring needles 7
- ☐ I get needles from the pharmacy 8
- ☐ I have been treated badly at needle exchange programs 9
- ☐ Friends/network partners have been treated badly at needle exchange programs 10
- ☐ I am worried I will lose my job or house if people find out I am an injection drug user 11

PROGRAMMER NOTE: Only a response from Q3 that is coded as 1 'YES' can be selected here

4. What is the name of the needle exchange program that you visited most recently?
   - ☐ Other 996

   4a. Was this a:
   - ☐ Government sponsored program 1
   - ☐ Private NGO 2
   - ☐ Other 996
   - ☐ Don't know 997
   - ☐ Refused 998

5. Why did you get your needles from the program you just mentioned?
   - ☐ The location is convenient (easy to get to) 1
   - ☐ They have other services (e.g., OST) there as well 2
   - ☐ I am treated well (with respect) at this program 3
   - ☐ My friends/network partners go there 4
   - ☐ Other 996
   - ☐ Don't know 997
   - ☐ Refused 998

INTERVIEWER INSTRUCTION: Ask question and let participant respond openly. Then fit answer into best option.

PROGRAMMER NOTE: Skip to Q7 for persons who did not inject drugs in the past 6 months**:** All of Q2c-Q9c=0 or missing).

6. In the last six months, how often did you get your syringes/needles from a… **[a-f]**?

	Never / Rarely	Less than half the time	Half of the time	More than half the time	Always
a. Needle Exchange Program	0	1	2	3	4
b. NGO	0	1	2	3	4
c. Pharmacy / Chemist	0	1	2	3	4
d. Hospital Dispensary	0	1	2	3	4
e. Friend	0	1	2	3	4
f. Dealer	0	1	2	3	4

INTERVIEWER INSTRUCTION for 6a-6f**:** Use **Card SV6**.
PROGRAMMER NOTE: Participant cannot choose "always" (4) or "more than half the time" (3) to more than one option.

- Repeat the question for each page a-f so for example 'In the last six months, how often did you get your syringes/needles from an Pharmacy/Chemist?'
- Include interviewer instruction on each page

INTERVIEWER READ: Now I am going to ask you about drug treatment. For now, I am only interested in opiate substitution programs such as methadone or buprenorphine. We will ask you about detoxification and other programs later.

7. When was the last time you participated in an opiate substitution program (e.g., Addnok, Methadone)?
   - ☐ Never 0 (→ Q8)
   - ☐ Within the last month 1 (→ Q10)
   - ☐ More than 1 month to 6 months ago 2 (→ Q10)
   - ☐ More than 6 months to 1 year (12 months) ago 3 (→ Q9)
   - ☐ More than 1 year to 2 years ago 4 (→ Q9)
   - ☐ More than 2 years ago to 4 years ago 5 (→ Q9)
   - ☐ More than 4 years ago 6 (→ Q9)
   - ☐ Don't know 997 (→ Q12)

INTERVIEWER INSTRUCTION: Ask the question and let participant respond openly. Then fit their answer into the best option.

8. Why have you never attended an opiate substitution program? (**SELECT ALL THAT APPLY**)

	Yes	No
8a. I inject very infrequently	1	0
8b. I do not need opiate substitution	1	0
8c. I do not know where to find an opiate substitution program	1	0
8d. The programs are too far away/ difficult to get to	1	0
8e. I do not have time to go	1	0
8f. The hours of operation of the programs were inconvenient for me	1	0
8g. None of my friends/network partners go	1	0
8h. Friends/network partners have been treated badly at opiate substitution programs	1	0
8i. I am worried I will lose my job or house if people find out I am an injection drug user	1	0
8j. Don't know	1 [12]	0
8k. Refused	1 [12]	0

INTERVIEWER INSTRUCTION: Please read all choices a-i to participant; Use **Card SV8.**

PROGRAMMER NOTE: Q8a-8k should be on one page

- If any of 'a-i' is selected at the same time as 'j' or 'k' then a pop-up box should appear 'You should not select DON'T KNOW/REFUSED when you have selected any other options. Please check answers.'

8a1. Which of these is the primary reason you have never attended an opiate substitution program?

- ☐ I inject very infrequently 1
- ☐ I do not need opiate substitution 2
- ☐ I do not know where to find an opiate substitution program 3
- ☐ The programs are too far away / difficult to get to 4
- ☐ I do not have time to go 5
- ☐ The hours of operation of the programs are inconvenient for me 6
- ☐ None of my friends/network partners go 7
- ☐ Friends/network partners have been treated badly at opiate substitution programs 8
- ☐ I am worried I will lose my job or house if people find out I am an injection drug user 9

PROGRAMMER NOTE: Skip to Q12.

- Only a response from Q8 that is coded as 1 'YES' can be selected here

9. Why have you not attended an opiate substitution program in the last six months?
(**SELECT ALL THAT APPLY**)

	Yes	No
9a. I have not injected in the past six months	1	0
9b. I do not need opiate substitution	1	0
9c. I do not know where to find an opiate substitution program	1	0
9d. The programs are too far away/ difficult to get to	1	0
9e. I do not have time to go	1	0
9f. The hours of operation of the programs were inconvenient for me	1	0
9g. None of my friends/network partners go	1	0
9h. I have been treated badly at opiate substitution programs	1	0
9i. Friends/network partners have been treated badly at opiate substitution programs	1	0
9j. I am worried I will lose my job or house if people find out I am an injection drug user	1	0
9k. Don't know	1 [10]	0
9l. Refused	1 [10]	0

INTERVIEWER INSTRUCTION for Q9**:** Please read all choices to participant; Use **Card SV9**.
PROGRAMMER NOTE : Q9a-9l should be on one page
- If any of 'a-j' is selected at the same time as 'k' or 'l' then a pop-up box should appear 'You should not select DON'T KNOW/REFUSED when you have selected any other options. Please check answers.'

9a1. Which of these is the primary reason you have not attended an opiate substitution program in the last six months?

- ☐ I have not injected in the past 6 months 1
- ☐ I do not need opiate substitution 2
- ☐ I do not know where to find an opiate substitution program 3
- ☐ The programs are too far away / difficult to get to 4
- ☐ I do not have time to go 5
- ☐ The hours of operation of the programs are inconvenient for me 6
- ☐ None of my friends/network partners go 7
- ☐ I have been treated badly at opiate substitution programs 8
- ☐ Friends/network partners have been treated badly at opiate substitution programs 9
- ☐ I am worried I will lose my job or house if people find out I am an injection drug user 10

PROGRAMMER NOTE: Only a response from Q9 that is coded as 1 'YES' can be selected here

10. The last time you participated in an opiate substitution program, which kind of program was it?

- ☐ Buprenorphine / Addnok 1
- ☐ Methadone 2
- ☐ Both 3
- ☐ Other 996
- ☐ Don't Know 997
- ☐ Refused 998

11. What is the name of the substitution therapy program that you attended most recently?

- ☐ Other 996

11a. Was this a:

- ☐ Government sponsored program 1
- ☐ Private NGO 2
- ☐ Other 996
- ☐ Don't know 997
- ☐ Refused 998

11b. Why did you choose this program?

- ☐ The location is convenient (easy to get to) 1
- ☐ They have other services (e.g., needle exchange) there as well 2
- ☐ I am treated well (with respect) at this program 3
- ☐ My friends/network partners go there 4
- ☐ Other 996
- ☐ Don't know 997
- ☐ Refused 998

INTERVIEWER INSTRUCTION: Ask the question and let participant respond openly. Then fit their answer into the best option.

12. Now I want to ask you about other types of drug treatment (aside from opiate substitution). Which other types of treatment have you received in the last six months? (**SELECT ALL THAT APPLY**)

	Yes	No
12a. Been in a hospital for at least one night	1	0
12b. Outpatient or drug-free treatment	1	0
12c. Detoxification	1	0
12d. Narcotics Anonymous or Alcoholics Anonymous	1	0
12e. Prayers or special religious practices	1	0
12f. Herbal / non-allopathic treatments	1	0
12g. Other	1	0
12h. Don't Know	1	0
12i. Refused	1	0

INTERVIEWER INSTRUCTION: Please read all choices to participant.
PROGRAMMER NOTE: Q12a-12i should be on one page

- If option '12h or 12i' is selected then all previous options '12a-12g' should be set to missing

13. How do you get condoms?

- ☐ I don't use condoms 1
- ☐ Out of pocket expense 2
- ☐ Government centers 3
- ☐ NGOs 4
- ☐ Primary health centers 5
- ☐ Hotspot condom vending machines 6
- ☐ Other 996
- ☐ Don't know 997
- ☐ Refused 998

INTERVIEWER INSTRUCTION: If more than one source is selected, ask them to select the main source

14. How easy is it for you to get condoms?

- ☐ I have no access to condoms 1
- ☐ Difficult but I can get them if I need them 2
- ☐ Easy 3
- ☐ Very easy 4
- ☐ Don't know 997
- ☐ Refused 998

15. Please indicate how important each of the following services is to you:

	Not Important	Somewhat important	Very important	Essential	Not applicable	Don't Know
15a. HIV testing	0	1	2	3		997
15b. Viral hepatitis Testing (HBV,HCV)	0	1	2	3		997
15c. HIV treatment (ART)	0	1	2	3		997
15d. Viral hepatitis treatment	0	1	2	3		997
15e. TB diagnosis/treatment	0	1	2	3		997
15f. Treatment for sexually transmitted infections	0	1	2	3		997
15g. Free condoms	0	1	2	3		997
15h. Education/counseling	0	1	2	3		997
15i. Opiate substitution treatment.	0	1	2	3	4	997
15j. Needle Exchange	0	1	2	3	4	997
15k. Nutritional support	0	1	2	3		997
15l. Overdose emergency services	0	1	2	3	4	997
15m. Services for sexual partners/spouses	0	1	2	3	4	997

INTERVIEWER INSTRUCTION: Use **Card SV15**.

PROGRAMMER INSTRUCTION: Please include the question on each page.

16.Please tell me how available each of the following services is to you:

	Not available	Available but hard to access	Easily available	Not applicable	Don't know
16a. HIV testing	1	2	3		996
16b. Viral hepatitis Testing (HBV,HCV)	1	2	3		996
16c. HIV treatment (ART)	1	2	3		996
16d. Viral hepatitis Treatment	1	2	3		996
16e. TB diagnosis/treatment	1	2	3		996
16f. Treatment for sexually transmitted infections	1	2	3		996
16g. Free condoms	1	2	3		996
16h. Education/counseling	1	2	3		996
16i. Opiate substitution treatment	1	2	3	4	996
16j. Needle Exchange	1	2	3	4	996
16k. Nutritional support	1	2	3		996
16l. Overdose emergency services	1	2	3	4	996
16m. Services for sexual partners/spouses	1	2	3	4	996

INTERVIEWER INSTRUCTION: Use **Card SV16**.
PROGRAMMER INSTRUCTION: Please include the question on each page.

17. If you could have one center in your community provide all of these services to you, which center would it be?

___ ___ ___
[Range: 01-20, 996,997,998
INTERVIEWER INSTRUCTION: Use Card SV17
- Code 996 for Other; 997 for Don't Know; 998 for Refused

PROGRAMMER NOTE: Skip to Q17b if Q17=997 or 998

17a. Which of the following reasons represent why you would want this center scaled up?
(**SELECT ALL THAT APPLY**)

	**Yes**	**No**
17a1. Ideal location	1	0
17a2, Flexible working hours	1	0
17a3. MSM-friendly	1	0
17a4. IDU-friendly	1	0
17a5. My friends also go to this center	1	0
17a6. The quality of care is good at this center	1	0
17a7. Privacy	1	0
17a8. I do not have to wait in line for a long time at this center	1	0
17a9. Other	1	0

INTERVIEWER INSTRUCTION: Please read all options to participants.
PROGRAMMER NOTE: Q17a1-17a9 should all be on one page.

17b. Which of the following services would you want available at this center?
(**Please Rank the top 5 in order**)

17b1. HIV testing ___
17b2. Viral hepatitis testing ___
17b3. HIV treatment (Antiretroviral therapy) ___
17b4. Viral hepatitis treatment ___
17b5. TB treatment ___
17b. Treatment for sexually transmitted infections ___
17b6. Free condoms ___
17b7. Counseling for mental and social issues ___
17b8. Opiate substitution treatment ___
17b9. Needle exchange ___
17b10. Non-HIV healthcare ___
17b11. Social and medical referrals ___
17b12. Testing of spouses/ sexual partners ___
(Acceptable range for 17b1-17b12: 1-5)
INTERVIEWER INSTRUCTION: Use card SV17b

- Rank the top 5 services that the participant reports by using numbers 1 -5 and leave others blank

PROGRAMMER NOTE: It is possible that some of these answers will be blank

- Q17b1-17b12 should all be on one page
- 1,2,3,4,5 must be at least used once and can only be used once

18. In the past six months, which of the following places did you visit for medical care?
(**SELECT ALL THAT APPLY**)

	Yes	No
18a. None - I didn't get any medical care	1 [20]	0
18b. Private medical doctor (in stand-alone clinic)	1	0
18c. Private medical doctor (in hospital)	1	0
18d. NGO doctor	1	0
18e. Government hospital doctor	1	0
18f. Alternative / non-allopathic (ayurveda/siddha/etc)	1	0
18g. Pharmacist / Chemist	1	0
18h. Other	1	0
18i. Don't Know	1	0
18j. Refused	1	0

INTERVIEWER INSTRUCTION: Please read all choices to participant.

- **PROGRAMMER NOTE:** Q18a-18j should all be on one page
- If participant selects 18a then the rest of the options should be removed.
- If any of 'a-h' is selected at the same time as 'i' or 'j' then a pop-up box should appear 'You should not select DON'T KNOW/REFUSED when you have selected any other options. Please check answers.'

19. In the last six months, which of the following places did you visit most often for medical care?

- ☐ Private medical doctor (in stand-alone clinic) 1
- ☐ Private medical doctor (in hospital) 2
- ☐ NGO doctor 3
- ☐ Government hospital doctor 4
- ☐ Alternative / non-allopathic (ayurveda/siddha/jolachap) 5
- ☐ Pharmacist / Chemist 6
- ☐ Other 996

PROGRAMMER NOTE: Only a response from Q18 that is coded as 1 'YES' can be selected here

INTERVIEWER READ: People can have many different types of problems getting their medical care. Think of the reasons why you **may not have gotten** the medical care you needed or that was recommended for you. Please indicate "Yes" or "No" for all of the following reasons for why you **may not have gotten needed medical care in the last month.**

20. In the last month, why have you not gotten the medical care that you needed or that was recommended for you? (**SELECT ALL THAT APPLY**)

	**Reasons:**	**Yes**	**No**
20a.	I was unable to pay for medical care	1	0
20b.	I was not sure where to go to get medical care	1	0
20c.	I did not have transportation to medical care	1	0
20d.	The clinic's hours of operation were inconvenient for me	1	0
20e.	I was treated poorly at a clinic in the past	1	0
20f.	I did not want to be seen at a clinic	1	0
20g.	I do not trust doctors	1	0
20h.	I don't really care about taking care of myself at this time	1	0
20i.	I have too many household responsibilities	1	0
20j.	I was too drunk or high	1	0
20k.	Don't Know	1	0
20l.	Refused	1	0

INTERVIEWER INSTRUCTION: Please read all choices to participant; Use **Card SV20.**
PROGRAMMER NOTE: Q20a-20l should all be on one page

- If any of 'a-j' is selected at the same time as 'k' or 'l' then a pop-up box should appear 'You should not select DON'T KNOW/REFUSED when you have selected any other options. Please check answers.'

21. In the last 6 months, have you been incarcerated?

- ☐ No 0
- ☐ Yes 1
- ☐ Don't know 997
- ☐ Refused 998

The India Men's Health Initiative

Participant ID ___ ___ ___ ___ ___
Date ___ ___/___ ___/___ ___
Interviewer Initials ___ ___ ___

Baseline: Tuberculosis History (TB)

INTERVIEWER READ: The next few questions are about tuberculosis.

1. Has a doctor or nurse ever told you that you had Tuberculosis (TB) disease? By TB disease, I mean have you been sick with TB and not just had a positive Tuberculin Skin Test (TST)?
   - ☐ No 0 (END SURVEY)
   - ☐ Yes 1
   - ☐ Don't know 997 (END SURVEY)
   - ☐ Refused 998 (END SURVEY)

2. In what year were you diagnosed with TB disease? (Estimate okay) ___ ___ ___ ___

INTERVIEWER INSTRUCTION: Code 1900 for Don't Know. [Range: 1900, 1910-present year]

3. How were you diagnosed with TB? (**SELECT ALL THAT APPLY**)

	**Yes**	**No**
3a. Sputum smear (saliva test)	1	0
3b. Chest X ray	1	0
3c. Blood Test	1	0
3d. Examination	1	0
3e. CT scan	1	0
3f. Other	1	0
3g. Don't Know	1	0
3h. Refused	1	0

PROGRAMMER NOTE: Q3a-3h should all be on one page.

- If any of 'a-f' is selected at the same time as 'g' or 'h' then a pop-up box should appear 'You should not select DON'T KNOW/REFUSED when you have selected any other options. Please check answers.'

4. Were you treated for your TB disease?
   - ☐ No 0 (END SURVEY)
   - ☐ Yes 1
   - ☐ Don't know 997 (END SURVEY)
   - ☐ Refused 998 (END SURVEY)

5. When were you treated for your TB disease?
   - ☐ Within the last month 1
   - ☐ More than 1 month to 6 months ago 2
   - ☐ More than 6 months to 1 year (12 months) ago 3
   - ☐ More than 1 year to 2 years ago 4
   - ☐ More than 2 years ago to 4 years ago 5
   - ☐ More than 4 years ago 6
   - ☐ Don't know 997
   - ☐ Refused 998

INTERVIEWER INSTRUCTION: Ask question and let participant respond openly. Then fit answer into the best option

6. Where did you receive your treatment for TB? (**SELECT ALL THAT APPLY**)

	Yes	No
6a. Large private hospital	1	0
6b. Government hospital	1	0
6c. Government DOT center	1	0
6d. Treated as part of a research study	1	0
6e. NGO doctor treated me in NGO	1	0
6f. Private doctor in stand-alone clinic	1	0
6g. Pharmacist/chemist	1	0
6h. Other	1	0
6i. Don't know	1	0
6j. Refused	1	0

INTERVIEWER INSTRUCTION: If participant says "government", make sure to distinguish government DOT center and government hospital.

PROGRAMMER NOTE: Q6a-6j should all be on one page.

- If any of 'a-h' is selected at the same time as 'i' or 'j' then a pop-up box should appear 'You should not select DON'T KNOW/REFUSED when you have selected any other options. Please check answers.'

7. What types of medicines did you take?
   - ☐ Tablets 1
   - ☐ Tablets + Injections 2
   - ☐ Don't know 997
   - ☐ Refused 998

8. How long did you take your medicines for? ___ ___ ___ months [Acceptable range, 0-36, 997, 998]

INTERVIEWER INSTRUCTION: Code 997 for Don't Know, 998 for Refused.

The India Men's Health Initiative

Baseline Depression (PHQ)

Participant ID ___ ___ ___ ___ ___
Date ___ ___/___ ___/___ ___
Interviewer Initials ___ ___ ___

INTERVIEWER READ: I am going to now ask some questions about your health. Please tell me how often you have been bothered by any of the following problems.

Over the last two weeks, have often have you been bothered by any of the following problems?

	Not at all	Several days	More than half the days	Nearly every day
1. Little interest or pleasure in doing things	0	1	2	3
2. Feeling down, depressed, or hopeless	0	1	2	3
3. Trouble falling or staying asleep, or sleeping too much	0	1	2	3
4. Feeling tired or having little energy	0	1	2	3
5. Poor appetite or overeating	0	1	2	3
6. Feeling bad about yourself - or that you are a failure or have let yourself or your family down	0	1	2	3
7. Trouble concentrating on things, such as reading the newspaper or watching television	0	1	2	3
8. Moving or speaking so slowly that other people could have noticed? Or the opposite – being so fidgety or restless that you have been moving around a lot more than usual	0	1	2	3
9. Thoughts that you would be better off dead or of hurting yourself in some way	0	1	2	3

INTERVIEWER NOTE for Q1-9**:** Use **Card PHQ1.**
PROGRAMMER NOTE: Include question and interviewer instruction on all Q1-9.
PROGRAMMER NOTE: If the participant selected 1-3 on any of the above questions, ask question 10. If they selected 0 for all nine questions above, END SURVEY.

10. Thinking about the problems you reported as bothersome in the questions I just asked, how difficult have these problems made it for you to do your work, take care of things at home, or get along with other people?
    - ☐ Not difficult at all 0
    - ☐ Somewhat difficult 1
    - ☐ Very difficult 2
    - ☐ Extremely difficult 3

The India Men's Health Initiative

Participant ID ___ ___ ___ ___ ___
Date ___ ___/___ ___/___ ___
Interviewer Initials ___ ___ ___

Baseline Social Support (SS)

INTERVIEWER READ: People sometimes look to others for companionship, assistance, or other types of support. How often was each of the following kinds of support available to you **during the past 4 weeks** if you needed it?

	None of the time	A little of the time	Some of the time	Most of the time	All of the time	Refused
1. How often do you have someone to love and make you feel wanted?	1	2	3	4	5	998
2. How often do you have someone to help with daily chores (child care, buying food, preparing meals) if you were sick?	1	2	3	4	5	998
3. How often do you have someone to help you buy medicines?	1	2	3	4	5	998
4. How often do you have someone to help with transportation?	1	2	3	4	5	998
5. How often do you have someone to give you money if you needed it?	1	2	3	4	5	998

INTERVIEWER INSTRUCTION: Use **Card SS1**.

6. How many meals do you eat every day at a regular time?
- ☐ None 1
- ☐ One 2
- ☐ Two 3
- ☐ Three or more 4
- ☐ Don't know 997
- ☐ Refused 998

7. Who helps you take care of all your health needs such as reminds you to take your medications, fixes your doctor's appointment, etc? (**SELECT ALL THAT APPLY**).

	**Yes**	**No**
7a. No one (I do it myself)	1	0
7b. Partner / spouse	1	0
7c. Parents	1	0
7d. Children	1	0
7e. Other Family	1	0
7f. Friends	1	0
7g. Roommates/housemate	1	0
7h. NGO/CBO worker (outreach worker/counselor)	1	0
7i. Government hospital staff	1	0
7j. Other	1	0
7k. Don't Know	1	0
7l. Refused	1	0

PROGRAMMER NOTE: Q7a-7l should all be on one page.

- If 7a is selected then remove all other options.
- If any of 'a-j' is selected at the same time as 'k' or 'l' then a pop-up box should appear 'You should not select DON'T KNOW/REFUSED when you have selected any other options. Please check answers.'

The India Men's Health Initiative

Baseline: Stigma (SG)

Participant ID ___ ___ ___ ___ ___
Date ___ ___/___ ___/___ ___
Interviewer Initials ___ ___ ___

INTERVIEWER READ: The next few questions are about activities in your life, and how people treat you in those activities.

INTERVIEWER INSTRUCTION FOR ALL QUESTIONS: Where the word MSM is used below in the questions, you can substitute kothi, panthi, DD, gay or bisexual depending on how the participant identifies himself in Q2 of DG.

	**Enacted stigma index**	**Never**	**Rarely**	**Sometimes**	**Frequently**	**Refused**
1.	How often has a hospital worker mistreated you because you are MSM?	0	1	2	3	998
2.	How often have you been refused medical care or denied hospital services because you are MSM?	0	1	2	3	998
3.	How often have you been asked to stay away from children because you are MSM?	0	1	2	3	998
4.	How often have family members forced you to move out of your home because you are MSM?	0	1	2	3	998
5.	How often has someone threatened to hurt you physically because you are MSM?	0	1	2	3	998
6	How often have you been refused housing because people suspect that you are MSM?	0	1	2	3	998

INTERVIEWER INSTRUCTION for Q1-6: Use **Card SG1**

	**Vicarious stigma**	**Never**	**Rarely**	**Sometimes**	**Frequently**	**Refused**
7.	How often have you heard stories about people being mistreated by hospital workers because they are MSM?	0	1	2	3	998
8.	How often have you heard stories about people being mistreated when getting HIV testing because they are MSM?	0	1	2	3	998
9.	How often have you heard stories about people being mistreated when getting HIV treatment because they are MSM?	0	1	2	3	998
10.	How often have you heard stories about someone being refused care from their family when they were sick because they are MSM?	0	1	2	3	998
11.	How often have you heard stories about people being forced by family members to leave their home because they are MSM?	0	1	2	3	998
12.	How often have you heard stories about a village or community ostracizing someone because they are MSM?	0	1	2	3	998

INTERVIEWER INSTRUCTION for Q7-12: Use **Card SG1**

	**Felt normative stigma scale**	**No one**	**Very few people**	**Some people**	**Most people**	**Refused**
13.	In your community, how many people avoid visiting the homes of people who are MSM?	0	1	2	3	998
14.	In your community, how many people think that family members who are MSM have brought shame on their families?	0	1	2	3	998
15.	In your community, how many people think that people who are MSM should feel guilty about it?	0	1	2	3	998
16.	In your community, how many people think that people who are MSM are disgusting?	0	1	2	3	998
17.	In your community, how many people think that people who are MSM are paying for their karma or sins?	0	1	2	3	998

INTERVIEWER INSTRUCTION for Q13-17**:** Use **Card SG13**

	**Internalized stigma scale**	**Not at all**	**A little**	**A fair amount**	**A great deal**	**Refused**
18.	How much do you feel that you should avoid visiting people because you are MSM?	0	1	2	3	998
19.	How much do you feel that you have brought shame on your family because you are MSM?	0	1	2	3	998
20.	How much do you feel guilty about being MSM?	0	1	2	3	998
21.	How disgusting do you feel because you are MSM?	0	1	2	3	998
22.	How much do you feel that you are MSM because you are paying for your karma or sins?	0	1	2	3	998

INTERVIEWER INSTRUCTION for Q18-22**:** Use **Card SG18**

PROGRAMMER NOTE for Q1-22**:** List the Interviewer Instruction related to which card to use on each question page.

The India Men's Health Initiative

Baseline: Quality of Life (QL)

Participant ID ___ ___ ___ ___ ___
Date ___ ___/___ ___/___ ___
Interviewer Initials ___ ___ ___

INTERVIEWER READ: Now, I am going to ask you some questions about your quality of life. The following questions are about activities you might do during a typical day.

Please indicate the statements that best describe your own health state today by choosing one answer / amount for each question.

	No	Yes, limits a little	Yes, limits a lot	Refused
1. Does your health now limit you in walking more than 50-100 meters?	0	1	2	998
2. Does your health now limit you in bathing or dressing yourself?	0	1	2	998
3. Does your health now limit you in work or other regular activities?	0	1	2	998

INTERVIEWER INSTRUCTION for Q1-3**:** Use **Card QL1.**

PROGRAMMER NOTE: List the Interviewer Instruction about which card to use on each question page

	None	Yes, limited a little	Yes, limited a lot	Refused
4. How much are you limited in your daily activities by pain or discomfort?	0	1	2	998
5. How much are you limited in your daily activities by anxiety or depression?	0	1	2	998

INTERVIEWER INSTRUCTION for Q4-5**:** Use **Card QL4.**

PROGRAMMER NOTE: List the Interviewer Instruction about which card to use on each question page

The India Men's Health Initiative
BASELINE: Quality of Life (QL)

INTERVIEWER READ: To help people say how good or bad a health state is, we have drawn a scale (somewhat like a thermometer) on which the best state you can imagine is marked 100 and the worst state you can imagine is marked 0.

We would like you to indicate on this scale how good or bad your own health is today, in your opinion. Please do this by indicating which point on the scale indicates how good or bad your health state is today.

6. ___ ___ ___ [Range 0-100, 998]

INTERVIEWER INSTRUCTION: Use **Card QL6.**

INTERVIEWER INSTRUCTION: Fill in the number on the scale that the participant has selected.

- Code 998 for Refused.

PROGRAMMER NOTE: Just leave a blank for the interviewer to enter the number. The actual scale does not need to be on the screen. We will have a paper copy of the scale for participants to use.

Your own health state today:

Best imaginable health state

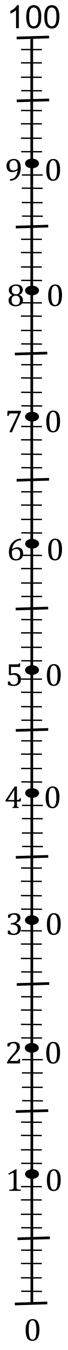

Worst imaginable health state

The India Men's Health Initiative

Baseline: Intervention Acceptability (IA)

Participant ID ___ ___ ___ ___ ___
Date ___ ___/___ ___/___ ___
Interviewer Initials ___ ___ ___

INTERVIEWER READ: I would like to ask you some questions about ways to prevent HIV infection.

1. Have you heard of the following as ways to prevent HIV infection? (**SELECT ALL THAT APPLY**)

	**Yes**	**No**
1a. Male Circumcision (Khatna)	1	0
1b. Vaginal/Rectal Microbicides	1	0
1c. Pre-Exposure Prophylaxis	1	0
1d. Treatment as Prevention	1	0
1e. Refused	1	0

INTERVIEWER INSTRUCTION: Please read all choices to participant.
PROGRAMMER NOTE: Q1a-1e should all be on one page

- If any of 'a-d' is selected at the same time as 'e' then a pop-up box should appear 'You should not select DON'T KNOW/REFUSED when you have selected any other options. Please check answers.'

Circumcision

2. Are you circumcised?

- ☐ No 0
- ☐ Yes 1 (→3c)
- ☐ Don't know 997
- ☐ Refused 998

3. There is now some evidence that circumcision (khatna) reduces the risk of a man picking up HIV infection from a woman. Would you be willing to be circumcised to reduce your risk of getting infected with HIV?

- ☐ No Chance 0 (→Q3b)
- ☐ Very little chance 1 (→Q3b)
- ☐ Some Chance 2
- ☐ Very good chance 3
- ☐ Undecided 4 (→Q3b)
- ☐ Refused 998 (→Q3b)

INTERVIEWER INSTRUCTION: Use card IA3.

3a. Why would you like to be circumcised (khatna)? (**SELECT ALL THAT APPLY**)

	**Yes**	**No**
3a1. HIV/STI prevention	1	0
3a2. Penile hygiene	1	0
3a3. Improved sexual pleasure	1	0
3a4. Lower risk of penile cancer	1	0
3a5. Lower risk of cervical cancer in female partner(s)	1	0
3a6. Better penile appearance	1	0
3a7. Other	1	0
3a8. Don't Know	1	0
3a9. Refused	1	0

INTERVIEWER INSTRUCTION: Please read all choices a1-a7 to participant.
PROGRAMMER NOTE: Q3a1-Q3a9 should all be on one page.

- If any of 'a-k' is selected at the same time as 'l' or 'm' then a pop-up box should appear 'You should not select DON'T KNOW/REFUSED when you have selected any other options. Please check answers.'
- Skip to Q4

3b. Why would you not be willing to be circumcised (khatna)? (**SELECT ALL THAT APPLY**)

	Yes	No
3b1. Fear of injury	1	0
3b2. Fear of pain	1	0
3b3. Against religious beliefs	1	0
3b4. I'm not at risk for HIV	1	0
3b5. Reduced sexual pleasure	1	0
3b6. Stigma/Discrimination	1	0
3b7. Fear that circumcision may cause infertility	1	0
3b8. I don't have sex with women	1	0
3b9. Other	1	0
3b10. Don't Know	1	0
3b11. Refused	1	0

INTERVIEWER INSTRUCTION: Please read all choices b1-b9 to participant.
PROGRAMMER NOTE: Q3b1-Q3b11 should all be on one page.

- If any of 'b1-b9' is selected at the same time as 'b10' or 'b11' then a pop-up box should appear 'You should not select DON'T KNOW/REFUSED when you have selected any other options. Please check answers.'
- Skip to Q4

3c. Why were you circumcised (khatna)? (**SELECT ALL THAT APPLY**)

	Yes	No
3c1. HIV/STI prevention	1	0
3c2. Penile hygiene	1	0
3c3. Improved sexual pleasure	1	0
3c4. Lower risk of penile cancer	1	0
3c5. Lower risk of cervical cancer in female partner(s)	1	0
3c6. Better penile appearance	1	0
3c7. Religious reasons	1	0
3c8. Medical reasons (doctor asked me to get it)	1	0
3c9. At Birth	1	0
3c10. Other	1	0
3c11. Don't Know	1	0
3c12. Refused	1	0

INTERVIEWER INSTRUCTION: Please read all choices c1-c10 to participant.
PROGRAMMER NOTE: Q3c1-Q3c12 should all be on one page.

- If any of 'c1-c10' is selected at the same time as 'c11' or 'c12' then a pop-up box should appear 'You should not select DON'T KNOW/REFUSED when you have selected any other options. Please check answers.'

PrEP

PROGRAMMER NOTE: Ask Questions 4-5a only if HIV status does not = 1 from Q8 or Q9 on **HIV** questionnaire. If HIV status = 1 (positive), skip to Question 6.

4. There is now some evidence that people who are not infected with HIV could take an HIV treatment pill daily to reduce their risk of becoming infected. Would you be willing to take an HIV treatment pill every day to reduce your risk of getting infected with HIV?

- ☐ No Chance 0
- ☐ Very little chance 1
- ☐ Some Chance 2 (→Q5)
- ☐ Very good chance 3 (→Q5)
- ☐ Undecided 4
- ☐ Refused 998

INTERVIEWER INSTRUCTION: Use card IA3.

4a. Why would you not be willing to take an HIV treatment pill every day? (**SELECT ALL THAT APPLY**)

	Yes	No
4a1. Side effects	1	0
4a2. Worry that the treatment won't work	1	0
4a3. Diet and sleep might be interrupted	1	0
4a4. Drug resistance might develop	1	0
4a5. People might think I have HIV/AIDS	1	0
4a6. Cost	1	0
4a7. It is a hassle to take a pill every day	1	0
4a8. I am not at risk for HIV	1	0
4a9. Other	1	0
4a10. Don't Know	1	0
4a11. Refused	1	0

INTERVIEWER INSTRUCTION: Please read all choices a1-a9 to participant.
PROGRAMMER NOTE: Q4a1-Q4a11 should all be on one page.

- If any of 'a1-a9' is selected at the same time as 'a10' or 'a11' then a pop-up box should appear 'You should not select DON'T KNOW/REFUSED when you have selected any other options. Please check answers.'

Microbicides

5. If there was evidence that use of gels or lubricants applied vaginally or rectally could reduce a person's risk of HIV or other STIs from sex, would you be willing to apply or have your partner apply a vaginal/rectal gel or lubricant to reduce your risk of getting infected with HIV?

- ☐ No Chance 0
- ☐ Very little chance 1
- ☐ Some Chance 2 (→Q6)
- ☐ Very good chance 3 (→Q6)
- ☐ Undecided 4
- ☐ Refused 998

INTERVIEWER INSTRUCTION: Use card IA3.

5a. Why would you not be willing to use gels or lubricants? (**SELECT ALL THAT APPLY**)

	Yes	No
5a1. Side effects	1	0
5a2. Worry that the gel/lubricant won't work	1	0
5a3. Drug resistance might develop	1	0
5a4. People might think I have HIV/AIDS	1	0
5a5. Cost	1	0
5a6. It is a hassle to apply a gel/lubricant every day before and after sex	1	0
5a7. My partner wouldn't like it if I used a gel/lubricant	1	0
5a8. May interfere with sexual pleasure	1	0
5a9. Other	1	0
5a10. Don't Know	1	0
5a11. Refused	1	0

INTERVIEWER INSTRUCTION: Please read all choices a1-a9 to participant.
PROGRAMMER NOTE: Q5a1-Q5a11 should all be on one page.

- If any of 'a1-a9' is selected at the same time as 'a10' or 'a11' then a pop-up box should appear 'You should not select DON'T KNOW/REFUSED when you have selected any other options. Please check answers.'

Early Treatment

PROGRAMMER NOTE: Ask Questions 6-6a only if HIV status = 1 (Positive) from Q8 or Q9 on **HIV** questionnaire.

6. The current Indian HIV treatment guidelines recommend treating someone when their CD4 drops to 350 cells/ul. There is now evidence that getting treated earlier (at higher CD4 cell counts) could prevent your sexual partner from getting HIV, and also improve your life at the same time. Would you be willing to start HIV medicines earlier?

- ☐ No Chance 0
- ☐ Very little chance 1
- ☐ Some Chance 2 (END SURVEY)
- ☐ Very good chance 3 (END SURVEY)
- ☐ Undecided 4
- ☐ Refused 998

INTERVIEWER INSTRUCTION: Use card IA3.

6a. Why would you not be willing to start treatment earlier? (**SELECT ALL THAT APPLY**)

	Yes	No
6a1. Side effects	1	0
6a2. Worry that the treatment won't work	1	0
6a3. Drug resistance might develop	1	0
6a4. It is a hassle to take medication	1	0
6a5. Cost	1	0
6a6. I feel healthy	1	0
6a7. I'm too busy	1	0
6a8. Other	1	0
6a9. Don't Know	1	0
6a10. Refused	1	0

INTERVIEWER INSTRUCTION: Please read all choices a1-a8 to participant.
PROGRAMMER NOTE: Q6a1-Q6a10 should all be on one page.

- If any of 'a1-a8' is selected at the same time as 'a9' or 'a10' then a pop-up box should appear 'You should not select DON'T KNOW/REFUSED when you have selected any other options. Please check answers.'

The India Men's Health Initiative Participant ID ___ ___ ___ ___ ___

Date ___ ___/___ ___/___ ___

Baseline: Sexual Health (SH) Interviewer Initials ___ ___ ___

INTERVIEWER INSTRUCTION: I am going to ask you some questions about sexual health. Some of these questions may be uncomfortable to answer. Please remember that we have not recorded your name and all answers will be confidential

1. Have you ever been told by a doctor, nurse or other medical provider that you have had…?

	**Yes**	**No**	**Don't know**	**Refused**
1a. Syphilis	1	0	997	998
1b. Gonorrhea	1	0	997	998
1c. Chlamydia	1	0	997	998
1d. Herpes Simplex	1	0	997	998
1e. Hepatitis A	1	0	997	998
1f. Hepatitis B	1	0	997	998
1g. Hepatitis C	1	0	997	998
1h. Trichomonas	1	0	997	998
1i. some other sexually transmitted infection	1	0	997	998

PROGRAMMER NOTE: Include question 'Have you ever been told by a doctor….' on each page

	a. In the last 6 months, did you have [**symptom**]?				b. In the last 6 months, did you seek medical care for [**symptom**]?			
	**Yes**	**No**	**Don't know**	**Refused**	**Yes**	**No**	**Don't know**	**Refused**
2. unusual genital or anal/rectal discharge	1	0 [3]	997 [3]	998 [3]	1	0	997	998
3. unusual genital or anal/rectal pain	1	0 [4]	997 [4]	998 [4]	1	0	997	998
4. a sore or ulcer in your genital or anal/rectal area	1	0 [5]	997 [5]	998 [5]	1	0	997	998

PROGRAMMER NOTE: If 2a ='1' and 2b~='1' or 3a = '1' and 3b ~='1' or 4a = '1' and 4b ~='1' then go to Q5

- If Q2a=0 and Q3a=0 and Q4a=0, go to Q6

5. In the last 6 months, why did you not seek care for these symptoms? (**SELECT ALL THAT APPLY**)

	Yes	No
5a. I did not know where to go	1	0
5b. The clinic where I would go is too far away / difficult to access	1	0
5c. I did not have time to go	1	0
5d. I did not have money to go	1	0
5e. I have been treated badly by doctors before at such clinics	1	0
5f. Friends /partners have been treated badly before at such clinics	1	0
5g. I do not consider these symptoms as a reason to go to a doctor	1	0
5h. Other	1	0

INTERVIEWER INSTRUCTION: Please read all choices to the participant.
PROGRAMMER NOTE: Q5a-5h should all be on one page.

5a1. Which of these is the primary reason you did not seek care when you had these symptoms?

- ☐ I did not know where to go 1
- ☐ The clinic where I would go is too far away / difficult to access 2
- ☐ I did not have time to go 3
- ☐ I did not have money to go 4
- ☐ I have been treated badly before by doctors at such clinics 5
- ☐ Friends / partners have been treated badly before at such clinics 6
- ☐ I do not consider these symptoms as a reason to go to a doctor 7
- ☐ Other 996

PROGRAMMER NOTE: Only a response from Q5 that is coded as 1 'YES' can be selected here

INTERVIEWER READ: The following questions are about any sexual abuse or violence you may have experienced in your life. Some of these questions may be uncomfortable to answer. Please remember that all of your responses will be kept confidential.

6. When you were growing up (before 16 years old), did you experience any serious physical violence? By physical violence, I mean were you ever hit, hit with an object, punched, kicked, or beaten up in a way that resulted in injury, severe pain, or other serious harm?
   - ☐ No 0
   - ☐ Yes 1
   - ☐ Don't Know 997
   - ☐ Refused 998

7. When you were growing up (before 16 years old), did you have any unwanted sexual experiences? By sexual experiences, I mean sexual touching or sexual intercourse, either oral or anal?
   - ☐ No 0
   - ☐ Yes 1
   - ☐ Don't Know 997
   - ☐ Refused 998

8. Has anyone (including a current or former spouse, boy/girlfriend, or other sexual partner) ever tried to make you have sex when you didn't want to?
   - ☐ No 0
   - ☐ Yes 1
   - ☐ Don't Know 997
   - ☐ Refused 998

9. Have you ever had a sexual partner (including a current or former spouse, boy/girlfriend, or other sexual partner) who has hit, slapped, kicked, pushed, shoved, or otherwise physically hurt you?
   - ☐ No 0
   - ☐ Yes 1
   - ☐ Don't Know 997
   - ☐ Refused 998

10. Have you ever talked to someone about having a surgery to change your sex (e.g. to become a TG)?
   - ☐ No 0
   - ☐ Yes 1
   - ☐ Don't Know 997
   - ☐ Refused 998

11. Has anyone ever tried to force you to have a surgery to change your sex (e.g., to become a TG)?
   - ☐ No 0
   - ☐ Yes 1
   - ☐ Don't Know 997
   - ☐ Refused 998

INTERVIEWER READ: The last few questions are about who you have talked to about your sexual preferences.

12. Have you told anyone that you are an MSM?
   - ☐ No 0 (→ Q14)
   - ☐ Yes 1

13. Who have you told that you are an MSM? (**SELECT ALL THAT APPLY**)

	**Yes**	**No**
13a. Spouse	1	0 [14]
13b. Regular female sex partner	1	0
13c. Parents	1	0
13d. Other family members	1	0
13e. Friends who are MSM	1	0
13f. Friends who are not MSM	1	0
13g. Coworkers	1	0
13h. Health care workers	1	0
13i. Counselors at an NGO/CBO	1	0

PROGRAMMER NOTE: Skip to Q15 except when 13a = 0 (No). If 13a = 0 (No), continue to Q14.

- If Q13a=1 then insert pop-up box saying 'Please double check the response. Then click Next to End the Survey.'

PROGRAMMER NOTE: Only ask Q14 if on DG, Q5 = 1 (Participant is married).

14. Why have you not told your spouse that you are an MSM? (**SELECT ALL THAT APPLY)**

	Yes	No
14a. Fear of rejection / abandonment / divorce	1	0
14b. Fear of psychological abuse	1	0
14c. Fear of physical abuse	1	0
14d. Fear of withdrawal of financial support	1	0
14e. Fear of not being able to be with children	1	0
14f. Fear of societal judgment	1	0
14g. MSM is a personal issue; want to keep it secret	1	0
14h. Did not think it was necessary or important	1	0
14i. Fear of stigma / discrimination	1	0
14j. Do not want to hurt wife	1	0
14k. Other	1	0
14l. Don't know	1	0

INTERVIEWER INSTRUCTION: Please read all choices to the participant.

- **PROGRAMMER NOTE:** If any of 'a-k' is selected at the same time as 'l' then a pop-up box should appear 'You should not select DON'T KNOW/REFUSED when you have selected any other options. Please check answers.'

15. What is the primary reason you have not told your spouse you are an MSM?

- ☐ Fear of rejection / abandonment / divorce 1
- ☐ Fear of psychological abuse 2
- ☐ Fear of physical abuse 3
- ☐ Fear of withdrawal of financial support 4
- ☐ Fear of not being able to be with children 5
- ☐ Fear of societal judgment 6
- ☐ MSM is a personal issue; want to keep it secret 7
- ☐ Did not think it was necessary or important 8
- ☐ Fear of stigma / discrimination 9
- ☐ Do not want to hurt wife 10
- ☐ Other 996

PROGRAMMER NOTE: Only an answer selected in Q14 can be selected in Q15.
